# Phosphorylation of the Peptidoglycan Synthase PonA1 Governs the Rate of Polar Elongation in Mycobacteria

**DOI:** 10.1371/journal.ppat.1005010

**Published:** 2015-06-26

**Authors:** Karen J. Kieser, Cara C. Boutte, Jemila C. Kester, Christina E. Baer, Amy K. Barczak, Xavier Meniche, Michael C. Chao, E. Hesper Rego, Christopher M. Sassetti, Sarah M. Fortune, Eric J. Rubin

**Affiliations:** 1 Department of Immunology and Infectious Disease, Harvard T. H. Chan School of Public Health, Boston, Massachusetts, United States of America; 2 Department of Microbiology and Physiological Systems, Howard Hughes Medical Institute, University of Massachusetts Medical School, Worcester, Massachusetts, United States of America; 3 Division of Infectious Disease, Massachusetts General Hospital, Boston, Massachusetts, United States of America; 4 Department of Microbiology and Immunobiology, Harvard Medical School, Boston, Massachusetts, United States of America; McGill University, CANADA

## Abstract

Cell growth and division are required for the progression of bacterial infections. Most rod-shaped bacteria grow by inserting new cell wall along their mid-section. However, mycobacteria, including the human pathogen *Mycobacterium tuberculosis*, produce new cell wall material at their poles. How mycobacteria control this different mode of growth is incompletely understood. Here we find that PonA1, a penicillin binding protein (PBP) capable of transglycosylation and transpeptidation of cell wall peptidoglycan (PG), is a major governor of polar growth in mycobacteria. PonA1 is required for growth of *Mycobacterium smegmatis* and is critical for *M*. *tuberculosis* during infection. In both cases, PonA1’s catalytic activities are both required for normal cell length, though loss of transglycosylase activity has a more pronounced effect than transpeptidation. Mutations that alter the amount or the activity of PonA1 result in abnormal formation of cell poles and changes in cell length. Moreover, altered PonA1 activity results in dramatic differences in antibiotic susceptibility, suggesting that a balance between the two enzymatic activities of PonA1 is critical for survival. We also find that phosphorylation of a cytoplasmic region of PonA1 is required for normal activity. Mutations in a critical phosphorylated residue affect transglycosylase activity and result in abnormal rates of cell elongation. Together, our data indicate that PonA1 is a central determinant of polar growth in mycobacteria, and its governance of cell elongation is required for robust cell fitness during both host-induced and antibiotic stress.

## Author Summary

Bacterial infections rely on continued bacterial growth. Studying cell growth is particularly important for pathogens such as *Mycobacterium tuberculosis* that grow differently than model organisms. Unlike *Escherichia coli* or *Bacillus subtilis*, which grow by incorporating cell wall material along their body, mycobacteria grow from the pole. It remains unclear how mycobacteria construct and extend their poles. Our work identifies a cell wall synthase, PonA1, as a key determinant of mycobacterial polar growth. PonA1 governs polar growth through two enzymatic activities that build the cell wall’s peptidoglycan (PG); both of these activities are required for normal cell growth. Changes in the amount or activity of PonA1 leads to misplaced cell poles and inhibition of cell proliferation. PonA1 is phosphorylated, an unusual modification for PG synthases. This phosphorylation tunes the rate of cell elongation. Changing PonA1’s regulatory or enzymatic activity impacts the survival of cells in the host or when treated with antibiotics. Our work shows how mycobacterial cell pole construction and cell fitness is governed by a major cell wall synthase; these findings may have implications for other bacteria that elongate from their poles.

## Introduction

*Mycobacterium tuberculosis*, the causative agent of tuberculosis (TB), remains a significant threat to human health. The World Health Organization estimates that 1.5 million individuals die of TB every year, and that roughly 2 billion people are latently infected with *M*. *tuberculosis* (*Mtb*)[1]. Despite the substantial global burden of TB, little is known about the bacterium’s basic physiological pathways, including cell growth and division.

Two processes govern growth of rod-shaped bacterial cells: elongation of the cell and then division of that cell into two daughter cells. This growth and division requires extensive remodeling of the cell wall, a layer or layers of complex saccharides that surrounds the cell’s plasma membrane. Cell division involves the synthesis and then splitting at mid-cell of a peptidoglycan layer (a macromolecule composed of disaccharides crosslinked by short peptides), called the septum. Bacteria elongate by incorporating new cell wall material into their existing cell wall. Many rod-shaped bacteria grow by elongating their mid-section; however, mycobacteria have a different body plan and elongate by incorporating new cell wall precursors at the cell poles[2,3].

Cell growth and division rely on the synthesis of new cell wall. The bacterial cell wall is required for cell survival and determines bacterial shape. Peptidoglycan (PG) is a major component of bacterial envelopes and is central to the integrity of the cell wall. PG is remodeled in response to stress and undergoes rounds of synthesis and hydrolysis every cell cycle to promote cell elongation and division[2,3]. Although coordination of PG synthesis has been extensively studied in other bacterial species, this process, particularly during elongation, remains relatively unexplored in mycobacteria.

One key enzyme that plays a central role in elongation and septation is PonA1, which localizes to the cell pole and septum, where it interacts with the essential PG hydrolase RipA[4,5]. Our previous work suggested that PonA1 was essential in *Mycobacterium smegmatis* (*Msm*)[4], indicating that we could use *Msm* as a convenient tool to define cellular activity specific to PonA1. Studying PonA1 enables us to determine how the cell governs one of the most critical steps in PG synthesis—elongation of the cell pole.

PonA1 is a penicillin binding protein (PBP), a member of a family of proteins that promotes cell growth and division through the synthesis of PG[2,3]. PonA1 has two extracellular catalytic domains that carry out the two necessary reactions for peptidoglycan synthesis: transglycosylation (TG) and transpeptidation (TP). TG reactions link the repeating disaccharide units that form the glycan backbone of peptidoglycan. Pentapeptide tails descend from the glycan chains and are crosslinked by TP reactions[2,3]. In addition to its two catalytic domains, PonA1 contains a long unconserved cytoplasmic tail. The cytoplasmic tail is phosphorylated[6], an unusual modification for PBPs that may play a role in PonA1’s cellular function.

Here we provide evidence that PonA1 is required for cell proliferation in *Msm* and is necessary for normal survival of *Mtb* during infection. It plays multiple roles in determining cell length and defining new growth. PonA1 is an early polar localizing factor that can nucleate elongation complexes to construct new cell poles, whose elongation rates are modulated by PonA1’s phosphorylation. Changes in PonA1 activity impact cell shape and growth, likely through insults to the cell wall peptidoglycan, which ultimately results in reduced cell fitness during infection and stress. Collectively, our data suggest that cell elongation in mycobacteria requires PonA1, whose catalytic and regulatory activities modulate the function of cell growth complexes.

## Results

### Normal growth of *M*. *smegmatis* and *M*. *tuberculosis* requires PonA1

**Fig 1:**
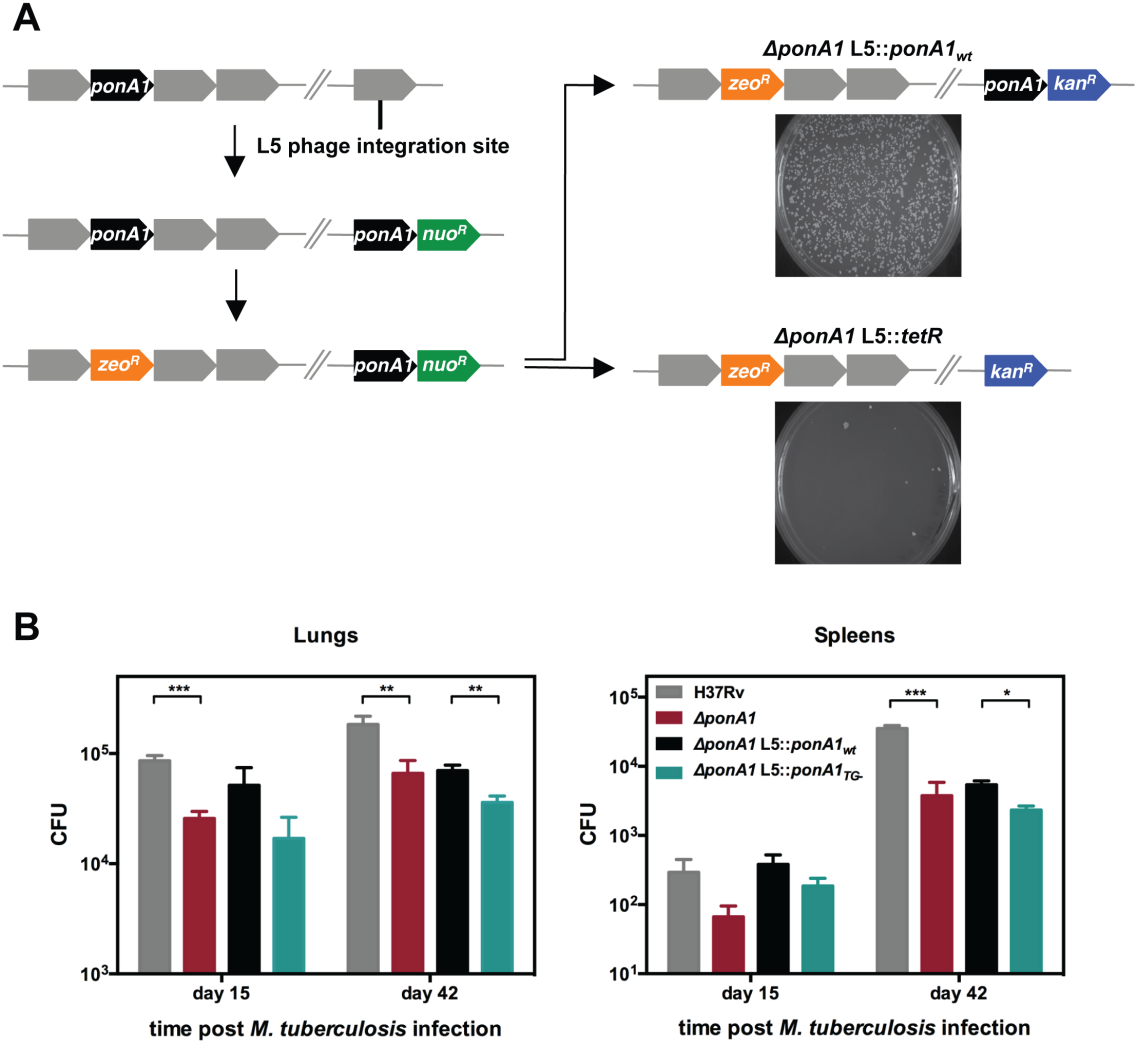
PonA1 is essential in *M*. *smegmatis* and required for normal growth of *M*. *tuberculosis*. **(A)** An allelic exchange system in *M*. *smegmatis* provides an efficient method to test the importance of PonA1 for bacterial survival. PonA1 is essential in *M*. *smegmatis*, as allelic exchange with a vector encoding *ponA1* complements bacterial growth, while allelic exchange with a negative control vector fails to rescue growth. **(B)** C57Bl6 mice were aerosol infected with H37Rv wildtype, *ΔponA1*, *ΔponA1*::*ponA1*_*wt*_, and *ΔponA1*::*ponA1*_*TG-*_ (an allele of PonA1 with a catalytic active site mutation in the transglycosylase (TG) domain), and CFU were enumerated were from lung and spleen homogenates at 15 and 42 days post infection (dpi). *ΔponA1* and *ΔponA1*::*ponA1*_*TG-*_ cells are less fit than H37Rv wildtype or isogenic wildtype, respectively, at 15 and 42 dpi. Statistical significance was calculated by a one-tailed t-test (lungs at 15 dpi, H37Rv compared to *ΔponA1* *** indicates p-value = 0.0005; lungs 42 dpi, H37Rv compared to *ΔponA1* ** indicates p-value = 0.0089, and *ΔponA1*::*ponA1*_*wt*_ compared to *ΔponA1*::*ponA1*_*TG-*_ ** indicates p-value = 0.0042. Spleens at 42 dpi, H37Rv compared to *ΔponA1* *** indicates p-value = 0.0001, and *ΔponA1*::*ponA1*_*wt*_ compared to *ΔponA1*::*ponA1*_*TG-*_ * indicates p-value = 0.0122). PonA1, while not required for growth of *M*. *tuberculosis* in culture, is required for normal bacterial multiplication and dissemination during infection in a mouse model of tuberculosis.

Transcriptional depletion of *ponA1* (MSMEG_6900, Entrez Gene ID 4536904) was previously shown to severely impact proliferation of *Msm* and induces the formation of lemon-shaped or ballooning cells, suggesting that PonA1 plays a critical role in cell growth in mycobacteria[4]. To determine whether PonA1 is essential for growth of *Msm*, we used an allelic replacement system[7] that allows exchange of PonA1 alleles on the bacterial chromosome (Fig 1A). We generated two exchange vectors, one encoding wildtype *ponA1* and a negative control vector lacking *ponA1*. Exchanging wildtype *ponA1* with a similar wildtype *ponA1* fully restored bacterial growth; however, exchange of *ponA1* with the negative control vector abolished bacterial growth (Fig 1A). The few remaining colonies on the negative control plate were confirmed *ponA1* ^+^ by PCR (seven colonies from approximately 4x10^8^ transformed cells). These data show that growth of *Msm* requires *ponA1*. However, transposon mutagenesis data from *M*. *tuberculosis* H37Rv (*Mtb*) suggested that *ponA1* (Rv0050, Entrez Gene ID 887065) could be disrupted without compromising proliferation of *Mtb* in culture[8], but that such a disruption would impact survival in a mouse model of TB[9]. We therefore tested whether *ponA1* could be deleted from the *Mtb* genome by recombineering (see Materials and Methods for details). We successfully isolated a *ΔponA1 Mtb* mutant and confirmed that *ΔponA1 Mtb* cells grew similarly to wildtype *Mtb* under standard laboratory culture conditions (S1A and S1B Fig). However, *ΔponA1 Mtb* cells replicated less robustly than wildtype *Mtb* in the lungs of C57Bl6 mice at 15 and 42 days post infection (dpi) and were moderately impaired in dissemination to the spleen at 42 dpi (Fig 1B), indicating that loss of PonA1 impacted *Mtb* fitness during infection. Together, these data show that PonA1 is necessary for normal growth of *Msm* in culture and promotes normal fitness of *Mtb* during infection.

### PonA1’s synthesis of glycan chains is essential for *M*. *smegmatis* viability and for normal cell length of *M*. *tuberculosis*

PonA1’s essentiality in *Msm* is unusual, since bifunctional PBPs in many bacterial species, including *Escherichia coli*[10], *Bacillus subtilis*[11], *Vibrio cholerae*[12], and even the closely related, polar growing actinomycete *Corynebacterium glutamicum*[13] have largely redundant roles. The *Msm* genome encodes two other bifunctional PBPs, PonA2 and PonA3, with presumably similar catalytic activities[14], whereas *Mtb* encodes just PonA1 and PonA2[15]. To identify which function of PonA1 was essential in *Msm*, we produced a panel of PonA1 mutants with varying catalytic functionality (S2A Fig) for use in the allelic exchange system shown in Fig 1A. Mutations in homologous active site residues eliminated catalytic activity in *in vitro* assays for *E*. *coli* PBP1a[16] and PBP1b[17]. In *E*. *coli*, genetic ablation of TP activity did not alter TG activity[16,17], whereas elimination of TG activity also abolished the enzyme’s TP activity *in vitro*, presumably because the substrate for the TP reaction was not produced[16,17]. However, whether loss of TP activity also abolishes TG activity in the cell remains unclear.

**Fig 2:**
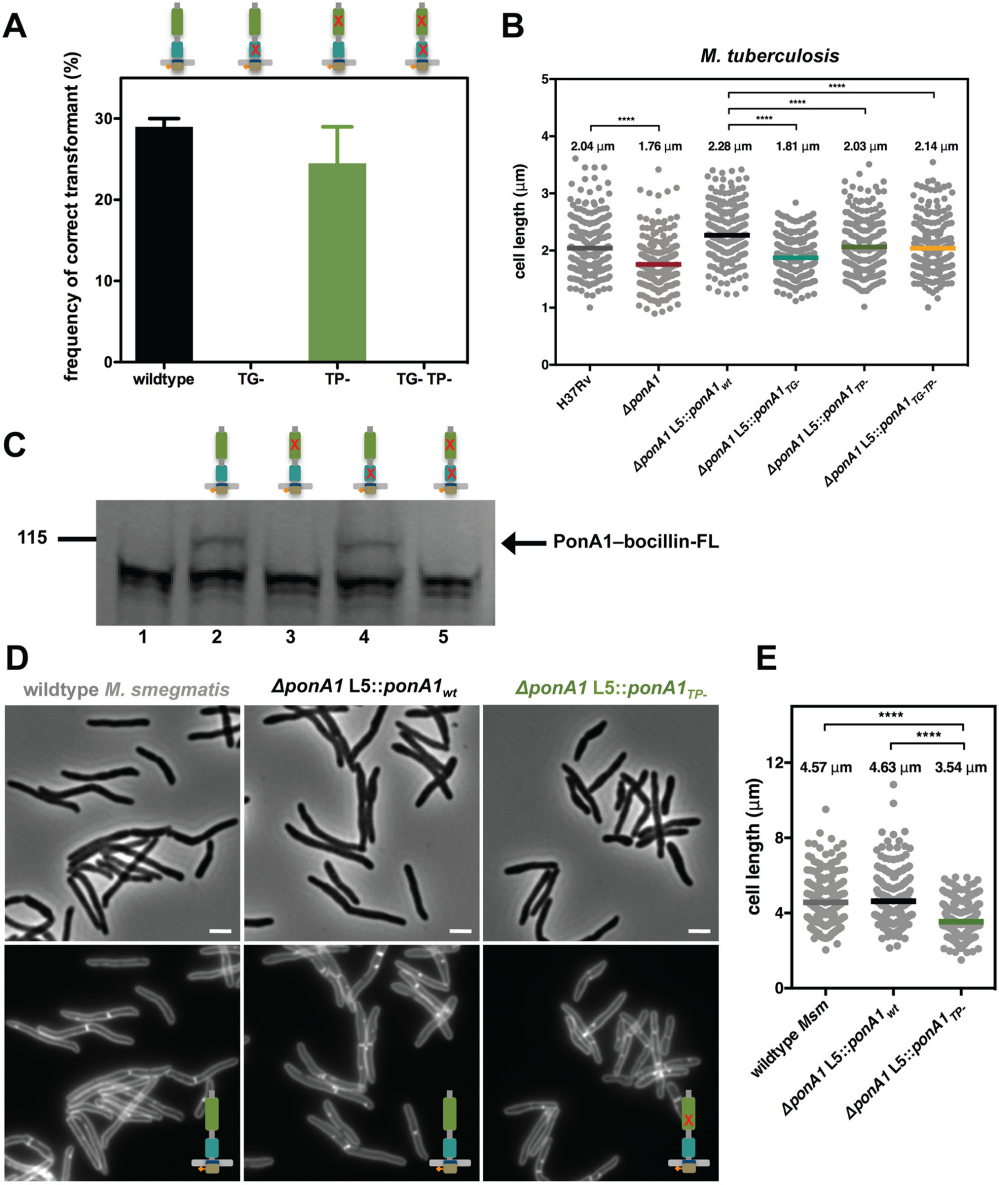
PonA1’s glycan synthesis is required in *M*. *smegmatis* and catalytic activity promotes normal cell elongation. **(A)** Allelic exchange (see Fig 1A) with vectors encoding alleles of PonA1 with varying catalytic activities (TG-, transglycosylase mutant; TP-, transpeptidase mutant; TG-TP-, transglycosylase and transpeptidase double mutant) demonstrates that a TG- allele fails to rescue bacterial survival. In contrast, a TP- allele complements bacterial growth. It is likely that PonA1’s synthesis of the glycan backbone of peptidoglycan is required for cell survival while other enzymes coordinate with PonA1 to crosslink those glycan strands into the existing sacculus. **(B)** *M*. *tuberculosis* strains were grown in culture and total cell length was measured: H37Rv wildtype (234 cells), *ΔponA1* (241 cells; approximate p-value < 0.0001 by the Kolmogorov-Smirnov test), *ΔponA1 L5*::*ponA1*_*wt*_ (212 cells), *ΔponA1 L5*::*ponA1*_*TG-*_ (220 cells; approximate p-value < 0.0001 by the Kolmogorov-Smirnov test), *ΔponA1 L5*::*ponA1*_*TP-*_ (227 cells; approximate p-value < 0.0001 by the Kolmogorov-Smirnov test), and *ΔponA1 L5*::*ponA1*_*TG-TP-*_ (209 cells; approximate p-value < 0.0001 by the Kolmogorov-Smirnov test). Loss of PonA1 or expression of a TG- allele of PonA1 reduces cell length compared to their isogenic wildtypes, supporting the role of PonA1’s TG activity in regulating cell growth in *M*. *tuberculosis*. Loss of TP activity had a more modest effect on cell length. **(C)** *Msm* PonA1 isoforms were produced in *E*. *coli* and assessed for their ability to bind bocillin-FL. Lane 1, negative control (wildtype *E*. *coli*); lane 2, PonA1_wt_; lane 3, PonA1_TP-_; lane 4, PonA1_TG-_; lane 5, PonA1_TG-TP-_. Mutants that lack TP activity cannot bind bocillin-FL. However, PonA1_TG-_ binds bocillin-FL at a level similar to wildtype PonA1, showing that PonA1’s catalytic activities can be uncoupled. **(D)** *Msm* cells that express PonA1_TP-_ do not exhibit gross cell shape changes, suggesting another enzyme coordinates peptidoglycan crosslinking with PonA1 to maintain cell integrity. Scale bar, 2 μm. **(E)** *ΔponA1 L5*::*ponA1*_*TP-*_ cells (229 cells) are shorter than wildtype *M*. *smegmatis* (wt Msm, 251 cells; approximate p-value < 0.0001 by the Kolmogorov-Smirnov test) or isogenic wildtype (*ΔponA1 L5*::*ponA1*_*wt*_, 247 cells; approximate p-value < 0.0001 by the Kolmogorov-Smirnov test), indicating that PonA1’s crosslinking influences cell length.

To test whether TG, TP or both activities were required to sustain growth, we transformed *Msm* with the exchange vectors and tested for appropriate chromosomal integration by antibiotic selection (see Materials and Methods). We found that only a PonA1_TP-_ allele (predicted to lack TP activity) was capable of rescuing bacterial growth while PonA1_TG-_ and PonA1_TG-TP-_ alleles (which lack either TG or both activities) failed to rescue bacterial survival (Fig 2A). These data suggest that PonA1’s polymerization of glycan strands is required in *Msm*, but that its crosslinking of PG is dispensable. This is consistent with a previous report of an *Msm* mutant with a transposon insertion in *ponA1* that generates a truncated protein that includes the TG domain but lacks the TP domain[18]. This mutant is likely still capable of transglycosylation but lost the ability to catalyze transpeptidation [18].

In *Mtb*, PonA1’s synthesis of glycan chains is required for normal bacterial growth. *Mtb* cells that expressed a PonA1_TG-_ allele exhibited moderately impaired viability and dissemination during infection, similar to *ΔponA1* cells, when compared to isogenic wildtype (Fig 1B). This fitness defect could be associated with abnormal cell length, as *ΔponA1* and *ΔponA1* L5::*ponA1*_*TG-*_ cells are shorter than their isogenic wildtypes by 14% and 21%, respectively (Fig 2B) but showed no gross cell shape changes or substantial population doubling defects (S1A, S1B and S1C Fig). The *ΔponA1* L5::*ponA1*_*TG-*_ cells also produced somewhat less phthiocerol dimycocerosate (PDIM) (S3 Fig), a cell wall lipid that is important for robust growth during infection. Lower PDIM production by this strain may contribute to the decreased fitness of these cells in the host, although we do not suspect that low PDIM is related to TG- PonA1 as low PDIM production has been described in unrelated genetic mutants[19]. Furthermore, *ΔponA1 Mtb* cells exhibit a cell length defect but produce PDIM at levels similar to or slightly above wildtype H37Rv, suggesting that PDIM loss is not correlated with decreased cell length. The change in cell length observed with *ΔponA1* and *ΔponA1* L5::*ponA1*_*TG-*_ cells suggests that PonA1’s polymerization of glycan chains is required for normal cell length in *Mtb*. Altered peptidoglycan structure in these shorter cells likely underlies their decreased ability to resist host-induced stresses.

### Correct cell length depends on PonA1’s crosslinking of peptidoglycan

The differential essentiality of PonA1’s TG and TP activities in the cell suggested that they could be uncoupled. Uncoupling of the TG and TP activities for PonA1 may be unusual. *in vitro* studies have shown that in other bifunctional PBPs, the TP domain crosslinks the nascent glycan strands produced by the *cis*-TG domain, and it cannot function in the absence of TG activity[16,17,20]. To determine whether PonA1’s crosslinking activity depended on its ability to polymerize glycan strands, we tested whether the PonA1_TG-_ allele could bind to a fluorescent penicillin analog, bocillin-FL, which covalently interacts with the transpeptidase domain active site serine[21]. Using FLAG-tagged PonA1 isoforms, we found that PonA1_TG-_ binds to bocillin at a level similar to wildtype PonA1, whereas PonA1_TP-_ and PonA1_TG-TP-_ did not bind to bocillin, as expected (Fig 2C). FLAG immunoblotting confirmed the bocillin-labeled band was PonA1-FLAG (S2B Fig). These data suggest that PonA1’s catalytic activities can be uncoupled, and specifically that its transpeptidase activity does not require active *cis*-transglycosylation.

Accordingly, we hypothesized that the uncoupling of PonA1’s catalytic activities could impact cell elongation and/or division, resulting in altered cell morphology. We imaged *Msm* cells that expressed PonA1_wt_ or PonA1_TP-_ and quantified cell length. We found that PonA1_TP-_ *Msm* were 24% shorter on average than isogenic wildtype (Fig 2D and 2E) and had impaired population doubling (S2C and S2D Fig) although similar levels of protein were expressed (S2E Fig). These data suggest that PonA1’s crosslinking of PG is required for normal cell length, and that the balance of PonA1 catalytic activities could have a role in controlling cell length and withstanding stress. Impaired PonA1 crosslinking in *Mtb* decreased cell length as well. Cells that expressed a PonA1_TP-_ or PonA1_TG-TP-_ allele were 11% or 6% shorter on average, respectively, than isogenic wildtype (Fig 2B). These data indicate that while PonA1’s TG activity is critical for cell viability and normal cell length (Figs 1B and 2), its TP activity also has a role in maintaining correct cell length in *Mtb*, although these mutations do not significantly alter population doubling rates (S1B, S1C and S1D Fig).

### PonA1’s transpeptidase domain is dispensable for cell survival

**Fig 3:**
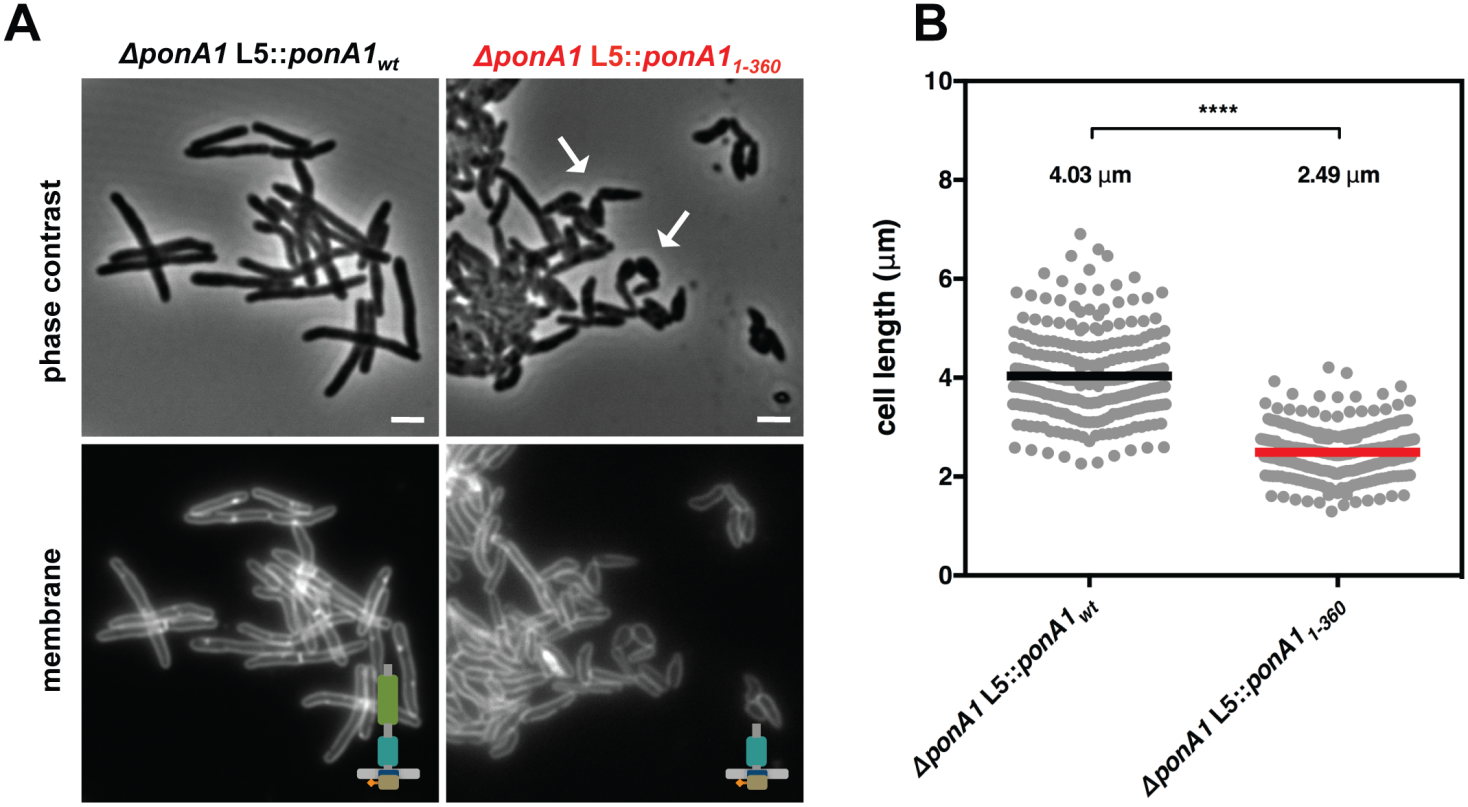
PonA1’s periplasmic domains modulate cell shape in mycobacteria. **(A)** Replacement of wildtype PonA1 with a PonA1 truncation containing the cytoplasmic tail and TG domain (PonA1_1-360_) rescues bacterial survival. Cells exhibit shape changes, but survive, suggesting that PonA1’s TG domain is the sufficient periplasmic domain required for bacterial growth. Cell shape may change due to missing protein-protein interactions that occur along the TP domain. Scale bar, 2 μm. **(B)** PonA1_1-360_ cells (266 cells) are shorter than isogenic wildtype (251 cells; approximate p-value < 0.0001 by the Kolmogorov-Smirnov test), which may indicate that the TP domain plays a role in complex formation that influences cell length. These measurements were taken on a different day than Fig 2C and 2D, hence the slight difference in isogenic wildtype cell length.

As PonA1’s TG activity is necessary for the viability of *Msm*, we hypothesized that the TG domain as the sole periplasmic domain would be sufficient to rescue bacterial growth. Furthermore, our previous work indicated that PonA1 dampened the hydrolytic activity of the RipA-RpfB complex *in vitro*[4], suggesting that PonA1’s modulation of RipA’s lytic activity could also contribute to the requirement of PonA1 in *Msm*. PonA1’s TP domain mediates the interaction with RipA[4]. To test whether *Msm* could survive with only PonA1’s TG domain in the periplasm, we generated a truncation mutant of PonA1 that eliminated the TP domain (PonA1_1-360_) (S2A Fig). We tested whether this truncation could complement loss of PonA1 via allelic exchange (Fig 1A). We found that PonA1_1-360_ could support bacterial survival and growth. However, cells that expressed PonA1_1-360_ exhibited cell shape defects (Fig 3A) and were 38% shorter than isogenic wildtype cells (Fig 3B). These morphological changes translated to diminished population growth rates (S2F and S2G Fig); however, these phenotypic differences were not due to difference in protein expression of the truncated PonA1 allele (S2H Fig). In sum, these data suggest that PonA1’s TG activity is necessary and that the TG domain is the critical periplasmic domain required for the survival of *Msm*. PonA1 modulation of RipA activity may be an important cellular role for PonA1, but it is not absolutely required for viability of *Msm*, which has other means of controlling RipA lytic activity[5].

### Linear polar elongation requires balanced PonA1 activity

**Fig 4:**
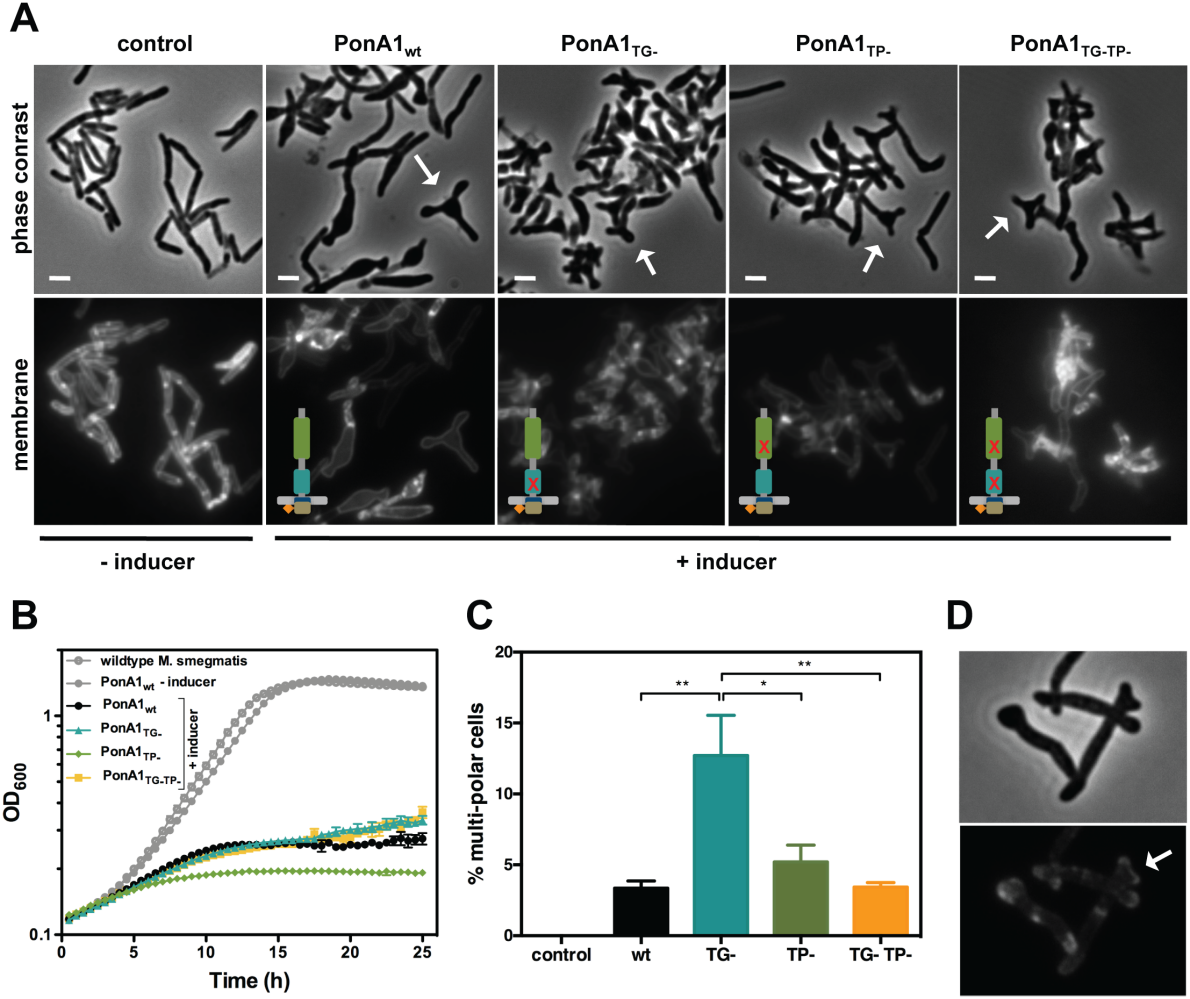
Excess PonA1 induces ectopic polar growth in *M*. *smegmatis*. **(A)** Overexpression of PonA1 causes the formation of ectopic growth poles, regardless of PonA1’s catalytic activity, suggesting that PonA1’s complex formation is sufficient to induce ectopic polar growth. Scale bar, 2 μm. **(B)** Excess levels of PonA1, including wildtype PonA1, inhibit bacterial multiplication, likely because of ectopic polar growth. **(C)** Although PonA1’s catalytic activity is not required for ectopic pole formation, it does influence the frequency at which ectopic poles form. Cells that express a TG- allele form ectopic poles at more than 2 times the frequency of other PonA1 alleles, suggesting that local peptidoglycan architecture could influence ectopic pole formation (statistical significance was assessed by one-way analysis of variance with Bonferroni’s multiple comparison test, and multiplicity adjusted p-values are reported. PonA1_wt_ compared to PonA1_TG-_, p-value = 0.0039; PonA1_TG-_ compared to PonA1_TP-_, p-value = 0.0225; PonA1_TG-_ compared to PonA1_TG-TP-_, p-value = 0.0042). **(D)** PonA1-RFP localizes to both growing tips of the ectopic pole, indicating that PonA1 is involved in nucleating elongation complexes at these growth tips.

Our data together suggest that PonA1 is critical for robust growth and that its catalytic activity is required for normal cell length. Do these critical catalytic activities lead to a dominant negative phenotype such that replacing wildtype PonA1 in cell growth complexes induces cellular toxicity? We tested this hypothesis by overproducing PonA1 in *Msm*. The overproduction of the PonA1_TG-TP-_ double mutant induced the formation of ectopic cell poles (Fig 4A) and severely inhibited population growth rates (Fig 4B), as did the single catalytic mutants. However, we observed the same phenotypes with overproduction of wildtype PonA1 (Fig 4A and 4B). This is in contrast to overproduction of the PonA1 homologue PBP1b in *E*. *coli*, which exhibits a dominant negative phenotype when overexpressed; namely, catalytically inactive PBP1b, but not wildtype PBP1b, inhibited growth and induced cell lysis[22]. These data suggest the cellular activity of PonA1 is different than its PBP1b homologue in *E*. *coli*. The growth inhibition with PonA1 overproduction in *Msm* correlated with the observation that the wildtype *ponA1* complement in *Mtb* (*ΔponA1* L5::*PonA1*_*wt*_), which is driven by a strong promoter, did not fully complement wildtype growth at later time points in both lungs and spleens (Fig 1B). These data suggest that overproduction of PonA1 in *Mtb* also impacted cell fitness. Collectively, these results indicate that the cell must tightly regulate PonA1 levels and activity to maintain robust cell growth.

Although PonA1’s enzymatic activity is not required for ectopic pole formation in *Msm*, it influenced the frequency of ectopic poles. Cells that overproduced PonA1_TG-_ induced the formation of multi-poled cells at a higher frequency than other alleles of PonA1 (Fig 4C). Taken together, these results suggest that some balance between TG and TP activities is required to induce pole formation. However, no enzymatic activity is required for this phenomenon, suggesting another role for PonA1, perhaps as a scaffold or recruitment factor within protein complexes that promote cell pole growth.

Previous work showed that ectopically-produced PonA1-RFP localized to the pole and septum[4]. A chromosomally RFP-tagged *ponA1* also exhibits polar and mid-cell localization (S3D Fig). To determine whether PonA1 localized at the ectopic growth pole, we imaged *Msm* cells that overproduced PonA1-RFP. We observed that PonA1-RFP localized to each ectopic tip of the growing pole (Fig 4D). Furthermore, PonA1 localized to the pole prior to its degeneration into ectopic poles (S5A Fig, follow pole with white arrow, and S1 Video), suggesting that PonA1 is an early localizing factor that promotes elongation of the cell pole. Ectopic polar growth occurred predominantly from one pole and not both poles (Figs 4A, S4 and S5), which correlates with the expected pattern of asymmetric polar elongation[23,24], and cells that did not overexpress PonA1 did not exhibit ectopic polar growth (Figs 4A, S5B and S2 Video). Occasional cells with bulges or ectopic poles exhibited no RFP signal at those foci. PonA1’s localization to the pole prior to the generation of ectopic poles indicates that PonA1 is intimately involved in not just elongating the pole, but in formation of the cell pole itself, potentially through interaction with DivIVA, which is required for pole formation in mycobacteria[24]. Furthermore, overexpression of PonA1 did not induce misplaced septa, suggesting that the majority of PonA1 is targeted to the cell pole during normal growth.

Ectopic polar growth upon overproduction of PonA1 in *Msm* is distinct from that observed in *E*. *coli*, where ectopic branches appeared when combinations of PBP1a and several low molecular weight PBPs were jointly deleted[25,26]. These data suggest that PonA1’s role in mycobacterial growth is distinct from the homologous bifunctional PBPs in *E*. *coli*. This phenotype may be due to the fundamentally different way that mycobacteria elongate—from the cell pole and not along the lateral body as in *E*. *coli*.

### Mycobacteria control cell elongation through post-translational modification of PonA1

**Fig 5:**
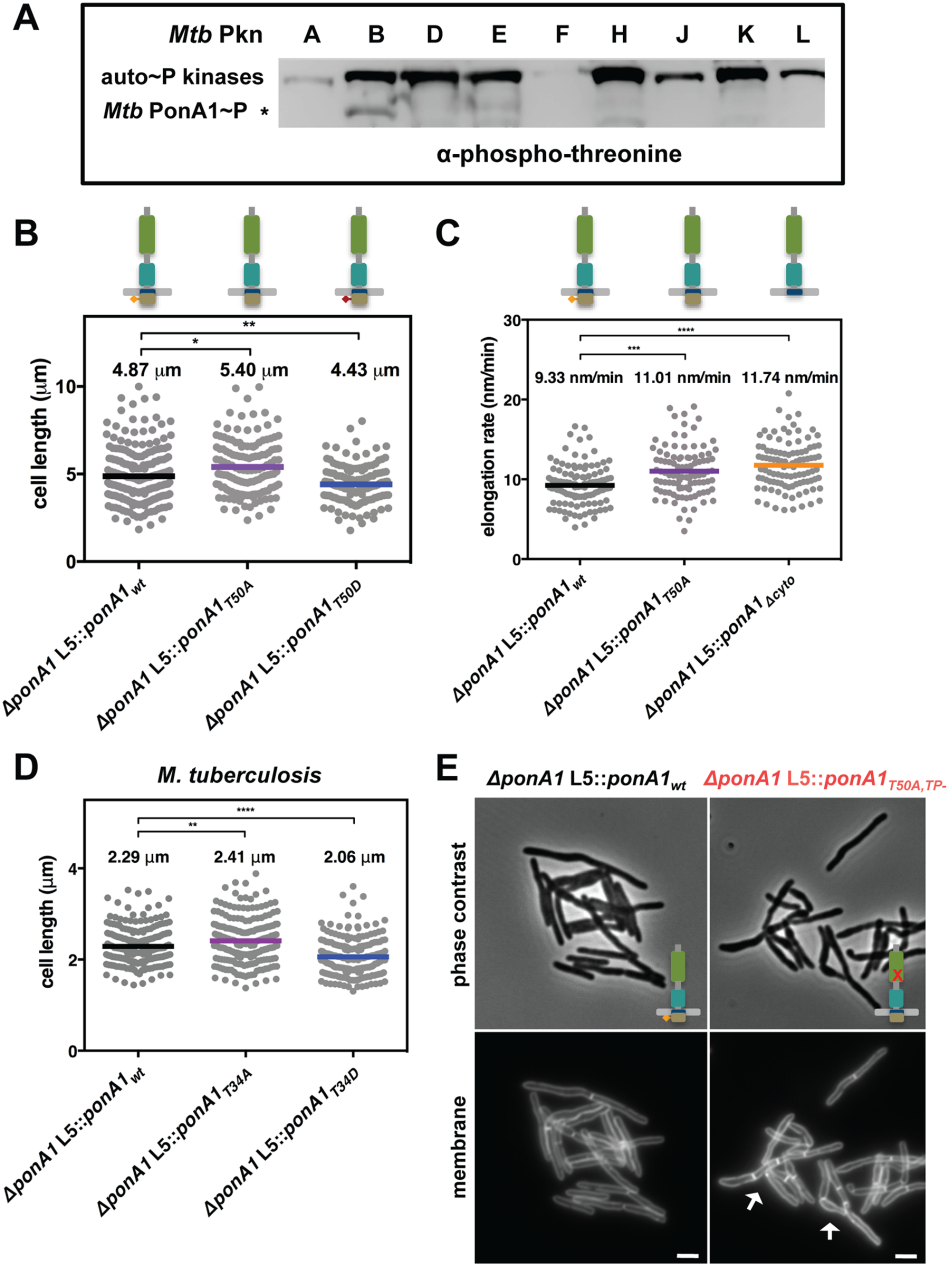
Phosphorylation regulates the rate of cell elongation. **(A)** Phospho-transfer profiling with the kinase domains of the major serine-threonine protein kinases of *M*. *tuberculosis* (*Mtb* Pkn) reveals that PknB efficiently phosphorylates *Mtb* MBP-PonA1_cyto_. **(B)** *M*. *smegmatis* cells that express a T50A allele of *ponA1* (134 cells; approximate p-value = 0.0145 by the Kolmogorov-Smirnov test) are longer than isogenic wildtype cells (219 cells), while cells that express a T50D allele (139 cells; approximate p-value = 0.0082 by the Kolmogorov-Smirnov test) are shorter than isogenic wildtype, suggesting PonA1’s phosphorylation regulates cell elongation or division. **(C)** Timelapse microscopy revealed that cells that expressed a T50A (127 cells; approximate p-value = 0.0002 by the Kolmogorov-Smirnov test) allele elongated faster than isogenic wildtype cells (174 cells), which was phenocopied by a truncation of the cytoplasmic tail (*Δ*cyto; 202 cells; approximate p-value < 0.0001 by the Kolmogorov-Smirnov test). These data suggest that PonA1’s phosphorylation negatively regulates cell elongation. **(D)** Similarly, phosphorylation status of PonA1 affects total cell length in *M*. *tuberculosis*. Cells that expressed a T34A allele (211 cells; approximate p-value = 0.0066 by the Kolmogorov-Smirnov test) exhibited an average cell length 5% longer than isogenic wildtype (202 cells), and cells that expressed a T34D allele (207 cells; approximate p-value < 0.0001 by the Kolmogorov-Smirnov test) were 11% shorter than isogenic wildtype. This suggests that PonA1’s unusual phosphorylation negatively regulates cell elongation in *M*. *tuberculosis* as in *M*. *smegmatis*. **(E)** *Msm* cells that encode a T50A,TP- allele of PonA1 are defective for normal cell separation. These cells form short chains of cells with multiple septa (white arrows). These data suggest that PonA1’s phosphorylation may regulate PonA1 TG activity, the remaining functional catalytic activity for this allele, and that alterations to PonA1’s TG activity impact the cell’s peptidoglycan and consequent cleavage of that peptidoglycan.

One method the cell may use to regulate PonA1 activity is through post-translational modification. The cytoplasmic tail of PonA1 from *Mtb* was recently identified as a substrate for the serine-threonine protein kinase PknB[6]. Prisic *et al* suggested the H37Rv genome had a misannotated start site for *rv0050*, which was corroborated by another report that identified an alternative protein translation start site in the 5’ UTR of *ponA1*[27]. We have used a construct beginning 426 nucleotides upstream of the annotated start site, which captures the phosphorylation site, reported translational start site, and yields a predicted transmembrane domain[28] (S6A Fig), a necessary feature of all PBPs[29]. We accordingly aligned the mc^2^155 genome for *Msm* PonA1; the start site was shifted 126 nucleotides upstream, which aligned well with -426 *Mtb* PonA1 (S6A and S6B Fig). Furthermore, the -426 *Mtb* PonA1 complemented growth of *Msm* lacking endogenous PonA1, which demonstrated that the -426 *Mtb* PonA1 allele was functional (S6A and S6B Fig). The -426 *Mtb* PonA1 protein was also produced at equivalent levels to *Msm* PonA1 (S6C Fig). To test whether -426 *Mtb* PonA1 was phosphorylated, we purified from *E*. *coli* the *Mtb* PonA1 cytoplasmic domain tagged to MBP along with his-MBP-tagged kinase domains of all the functional serine-threonine kinases of *Mtb*. Phospho-transfer profiling of MBP-*Mtb* PonA1_cyto_ with the kinases revealed that PknB efficiently phosphorylated -426 *Mtb* PonA1 *in vitro* (Fig 5A). The specificity of the phosphorylation on MBP-*Mtb* PonA1_cyto_’s T34 residue (S6B Fig) was verified by mass spectrometric analysis (S7D Fig).

Because PknB’s phosphorylation of other targets modulates key steps in cell growth and division[2], we hypothesized that PonA1’s phosphorylation might play a role in regulating cell elongation or division. We tested this hypothesis by constructing two different alleles of *Msm* PonA1, one in which phosphorylation is blocked by substitution of an alanine residue for the targeted threonine (T50A) and another where an aspartate substitution might mimic the effects of phosphorylation (T50D) and confirmed these isoforms were stable (S6C Fig). We swapped these for the native allele and measured *Msm* cell length. Cells that expressed the T50A allele were longer than isogenic wildtype, whereas cells that expressed the T50D allele were shorter than isogenic wildtype (Fig 5B). Because the observed changes in cell length did not alter gross optical density measurements of these cell populations (S8A Fig), we used timelapse microscopy to measure single cell elongation and division events. Cells were labeled with a green fluorescent amine-reactive dye that stains the cell surface and does not diffuse over time. This allows the visualization of new cell growth (S8B Fig)[23,30]. After labeling, cells were imaged in custom microfluidic devices, and cell elongation and cell division events were quantified over time (S8B Fig; see Materials and Methods for details). *Msm* cells that expressed PonA1_T50A_ allele elongated 15% faster than isogenic wildtype (Fig 5C). This faster elongation rate correlated with increased cell length at division (S8C Fig), and did not alter generation time at the single cell level (S8D Fig). Timelapse microscopy analysis did not show substantial growth differences between the PonA1_T50D_ mutant and isogenic wildtype cells. Truncation of the cytoplasmic tail of PonA1 (PonA1_Δcyto_), which generated a stable protein (S6C Fig), phenocopied the increased elongation rate (Fig 5C), supporting the role of PonA1’s cytoplasmic domain, and likely its phosphorylation, in downregulating cell elongation. Cells that expressed PonA1_T50A_ or PonA1_Δcyto_ elongated predominantly from the old pole (S8E Fig) instead of the new pole (S8F Fig), in agreement with the expected growth pattern for mycobacteria[23,24]. These data indicate that loss of PonA1’s phosphorylation does not alter the subcellular localization of elongation complexes or PonA1’s localization in the elongation complex itself. Thus, it appears that PonA1’s cytoplasmic domain is not required for localization of PonA1 in the elongation complex or for PonA1’s function in that complex. However, PonA1’s cytoplasmic tail could interact with cytoplasmic factors that are involved in modulating or are required for the activity of the elongation complex. For example, it could mediate interaction with DivIVA, an essential determinant of polar growth in mycobacteria [24].

PonA1’s phosphorylation plays a similar role in *Mtb*. We measured the length of cells that expressed a PonA1 T34A or T34D isoform. We found that the T34A mutant increased cell length by 5% compared to isogenic wildtype, while *Mtb* that encoded the T34D allele were 11% shorter than isogenic wildtype (Fig 5D). These data correlate with the observation in *Msm*. Furthermore, expression of T34A *Mtb* PonA1 in *Msm* increased cell length of *Msm* while expression of T34D *Mtb* PonA1 decreased *Msm* cell length. Collectively, these data suggest that PonA1’s phosphorylation reduces elongation in both *Mtb* and *Msm* and provides the cell with a facile method to modulate cell length, an important response to certain stress conditions, including in-host survival[31].

Because PonA1’s phosphorylation was not essential for protein function, but did modulate cell elongation rate, we hypothesized that PonA1’s phosphorylation regulated its enzymatic activity. To test this, we generated a double mutant in which the phosphorylation and transpeptidase active sites were ablated. *Msm* cells that expressed this double PonA1 mutant (PonA1_T50A,TP-_) were defective for normal cell separation. Cells formed short chains with cells of mixed length containing several septa (Fig 5E). This is distinct from the short cell phenotype of TP- cells (Fig 2C and 2D) and the elongated cell phenotype of T50A cells (Fig 5B), neither of which form short chains. Additionally, the *Msm* PonA1_T50A,TP-_ cells had diminished growth rates compared to either of the single mutants or isogenic wildtype (S9A and S9B Fig), although similar protein levels were produced (S9C Fig). These data suggest that the peptidoglycan synthesized by this PonA1 mutant diminishes efficient cleavage. This could indicate that PonA1’s phosphorylation regulates its TG activity—the remaining enzymatic activity in this mutant—and that the observed division defects result from imbalanced synthesis of peptidoglycan.

### Altering PonA1 activity changes antibiotic susceptibility

Because changes to PonA1’s phosphorylation status or synthetic ability altered cell shape and viability, we investigated whether these mutations would impact cell physiology under stress conditions, particularly antibiotic treatment. We previously demonstrated that single nucleotide polymorphisms in *ponA1* identified in clinical isolates alter *Mtb*’s fitness during rifampicin treatment[32]. We tested if altered *Mtb* PonA1 enzymatic or regulatory activity would also impact susceptibility to rifampicin. Indeed, *Mtb* cells that expressed PonA1_TG-_ or PonA1_T34D_ were 5 and 4 fold more tolerant to rifampicin treatment than isogenic wildtype, respectively (S10A Fig). In contrast, the inactivation of PonA1’s TP activity or blockade of phosphorylation did not alter cell survival in the presence of rifampicin (S10A Fig). In sum, these data suggest that altering PonA1 catalytic and regulatory activity changes cell fitness in the presence of the clinically relevant antibiotic rifampicin, although the mechanism remains unclear.

**Fig 6:**
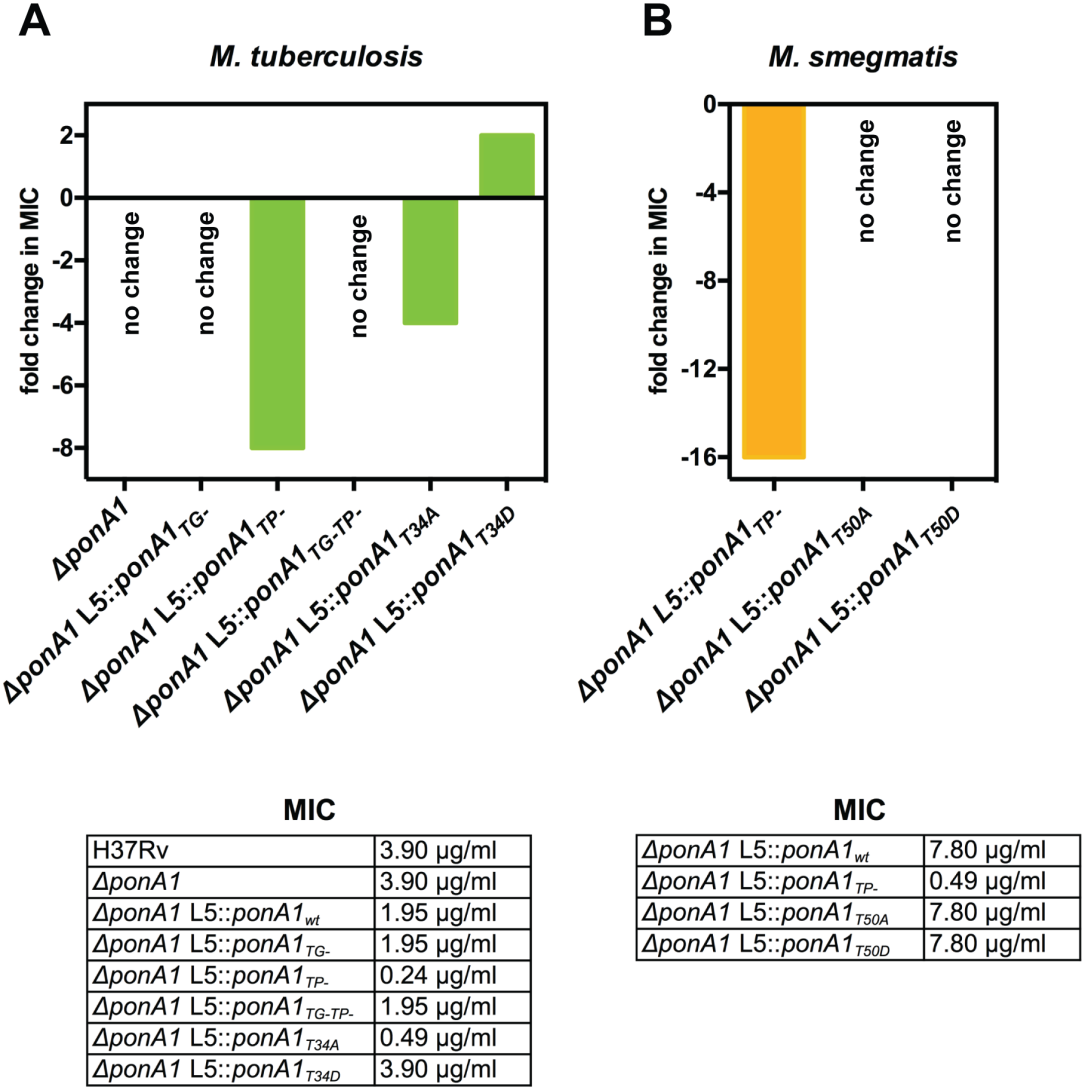
PonA1’s transpeptidase activity is required for normal teicoplanin tolerance in mycobacteria. **(A)** Minimum inhibitory concentration (MIC) of teicoplanin for wildtype and different PonA1 mutant *M*. *tuberculosis* strains is tabulated. Fold change in MIC is calculated from wildtype *Mtb* (for *ΔponA1*) or isogenic wildtype (for the TG-, TP-, TG-TP-, T34A, and T34D strains). Cells that express a PonA1 TP mutant or the T34A allele are more sensitive to teicoplanin than isogenic wildtype. No change, no fold change in MIC from appropriate wildtype strain. **(B)** Teicoplanin MICs for *M*. *smegmatis* mutant strains are tabulated. Fold change is calculated from isogenic wildtype. Cells that express a PonA1 TP mutant are more sensitive to teicoplanin than isogenic wildtype.

We also hypothesized that changes in PonA1 activity would alter susceptibility to cell wall stress, including antibiotics that target PG synthesis. Genetically ablating *Mtb* PonA1’s TP activity or its phosphorylation site decreased by 8- or 4-fold, respectively, the minimum inhibitory concentration (MIC) of teicoplanin, a glycopeptide that inhibits PG crosslinking[33] (Fig 6). The increased sensitivity to teicoplanin suggests that imbalanced peptidoglycan synthesis occurred in TP- as well as T34A cells, supporting a model in which phosphorylation may regulate PonA1’s TG activity (the remaining catalytic activity of the TP- mutant). A shifted teicoplanin MIC was also observed in *Msm* where the TP- allele exhibits a 16-fold change in MIC (Fig 6), although the *Msm* T50A allele did not phenocopy the shifted teicoplanin MIC observed in *Mtb*. However, *Msm* cells that expressed PonA1_T50A_ alleles were more susceptible to a range of meropenem concentrations (S10B Fig), supporting the idea that balanced PonA1 activity is required for architecturally sound peptidoglycan to resist stress. We also tested whether inhibiting glycan synthesis would impact cellular fitness of PonA1 mutants, but observed minimal efficacy of the small molecule inhibitor moenomycin A (S10C Fig).

Our data suggested that PonA1 activity modulated cell fitness in the presence of drugs. We tested whether changes to PonA1 activity would alter cell fitness under other chemical stresses that target the cell wall. Upon treatment with SDS, *Msm* cells that expressed PonA1_TP-_ and PonA1_T50A_ exhibited a modest decrease in survival (S11A and S11B Fig). D-amino acids have also been shown to modulate cell wall homeostasis[34], and we tested the fitness of PonA1_TP-_ cells in the presence of D-Met. *Msm* PonA1_TP-_ cells grew less robustly and did not reach the same optical density at stationary phase as compared to isogenic wildtype cells (S11C and S11D Fig). Furthermore, PonA1_TP-_ cells exhibited aberrant cell shape in deep stationary phase. After four days of growth in culture, *Msm* PonA1_TP-_ cells became wider and shorter compared to isogenic wildtype (S11E Fig, white arrows). These data together indicate that normal PonA1 catalytic and regulatory activity is required for the synthesis of structurally robust peptidoglycan to ensure cellular survival in the face of multiple stresses.

## Discussion

The paradigms of cell growth and division in rod-shaped bacteria are based on organisms that grow fundamentally differently than mycobacteria. Consequently, our understanding of mycobacterial growth—and the cell’s ability to repress growth—remains elementary. We have addressed this knowledge gap by investigating how a key cell wall synthase, PonA1, promotes and regulates cell growth in mycobacteria.

### PonA1 is non-redundant during cell growth

We found that PonA1 is essential in *Msm* and required for normal proliferation in the lung as well as dissemination outside of the lung during an *Mtb* infection (Fig 1). These data suggest that the major PBPs in mycobacteria do not have truly redundant functions. This is similar to *E*. *coli* where distinct complexes employ PBP1a or PBP1b in *E*. *coli*[3], as well as recent work in *V*. *cholerae*[12] and *Listeria monocytogenes*[35] that show PBP1a and PBP1b have specialized biological roles during infection. Furthermore, this implies a revision of the classic understanding of the PBPs as redundant factors. Imperfect redundancy during stress could be exploited to identify unique physiological roles for the PBPs in a variety of bacterial species.

The differential essentiality of PonA1 in *Msm* and *Mtb* for growth in culture is somewhat surprising; it suggests a difference in expression or functionality of other PBPs, such as the highly homologous protein PonA2, in these species. The role for bifunctional PBPs in *Mtb* cell elongation is probably jointly filled by PonA1 and PonA2, whereas *Msm* has evolved to solely depend on PonA1. Even though PonA1 exhibits different essentiality in *Mtb* and *Msm*, the role of PonA1 is likely highly similar in the two species. We found that *Mtb* PonA1 complements survival of *Msm* depleted of endogenous PonA1 (S7 Fig), and the two proteins are 70% identical at the sequence level (S6 Fig). Furthermore, our data suggest that the physiological role of PonA1 in both *Msm* and *Mtb* is very similar.

### Governance of cell length and shape

**Fig 7:**
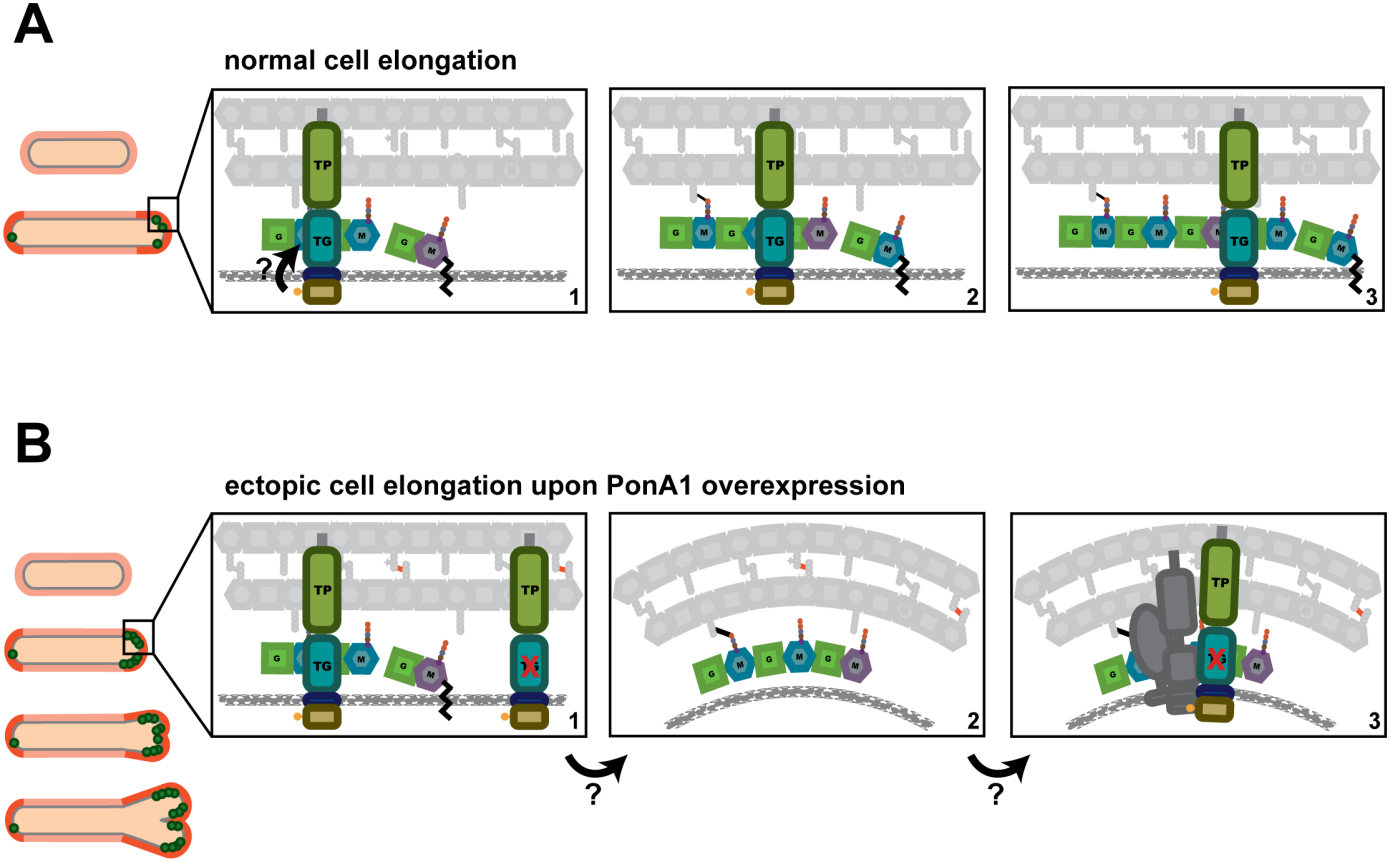
A model for how PonA1 promotes cell elongation in mycobacteria. **(A)** We propose a model wherein PonA1 localizes early to the growth tip and promotes pole elongation through PG synthesis. PonA1 may recruit other factors to form the elongation complex or they are recruited independently of PonA1 (factors not shown). Together with these factors, polar elongation proceeds (orange cells). PonA1 furthers cell elongation through its synthesis of PG. PonA1’s synthesis of glycan strands (colored subunits, panels 1–3) and its crosslinking of those glycan strands (black lines, panels 2–3) promote normal cell length. These catalytic activities are potentially modulated through PonA1’s phosphorylation (arrow), which acts to regulate the rate of cell elongation. **(B)** Excess PonA1 stimulates ectopic cell elongation (orange cells). The frequency of ectopic pole formation increases with imbalanced PG crosslinking and may result from changes in local peptidoglycan architecture (panels 1–2, factors removed from panel 2 for clarity). These architectural changes may act like a sink that ‘recruits’ additional protein-interactors of PonA1 or additional elongation complex components to spur cell elongation at ectopic sites (panel 3, dark gray factors).

Our data suggest that PonA1’s polymerization of glycan strands plays a key role in determining cell length (Figs 2 and 7A). Indeed, overproduction of TG-inactive PonA1 in *Msm* yields cells 38% shorter when compared to isogenic wildtype (Figs 4A, S4A and S4B). These cells’ length defect is not solely due to their higher frequency of ectopic pole formation, as even cells without ectopic poles are noticeably shorter than isogenic wildtype (S4A Fig). Balanced PonA1 catalytic activity, however, is required for normal cell length. Whereas TG seems to play a large role in modulating cell length, PonA1’s TP activity is also required for normal cell length (Fig 2). This supports the model wherein efficient PG synthesis occurs when one enzyme is catalytically capable of both synthesizing and crosslinking nascent glycan strands[3]. The linking of PonA1’s activities provides tight control over cell length and the protection of cell integrity and growth.

We found that PonA1-specific PG crosslinking is not required in mycobacteria, although it does promote normal cell length. This suggests that other factors either can compensate for lack of PonA1 crosslinking when absent or that PonA1 normally functions in the elongation complex with another crosslinking enzyme[2], as is true for bifunctional PBPs in other bacterial species[3,20]. Because mycobacterial peptidoglycan is estimated to contain predominantly 3–3 crosslinks[36,37] formed by l,d-transpeptidases, these ‘non-classical’ 3–3 crosslinking enzymes may be active in the elongation complex under wildtype conditions. Indeed, deletion of LdtB in *Mtb* results in shorter cells[38].

### Linear cell growth

We found that PonA1 catalytic activity is required for normal cell length and that PonA1 is an early acting factor that promotes extension of the cell pole. Overproduced PonA1-RFP localizes to the cell pole when the cell is still rod-shaped (S5 Fig). These cells then generate an ectopic pole, which is also marked by PonA1-RFP. We also see the formation of ectopic poles when PonA1-FLAG is overproduced (Figs 4 and S4), suggesting that this observation is not an artifact of the fusion partner. Combined with the fact that enzymatically inactive PonA1 induces ectopic pole formation, these data suggest that PonA1 is important for nucleating elongation complexes, and that PonA1 is a major factor that promotes pole elongation in mycobacteria. Because overproduction of enzymatically inactive PonA1 still results in ectopic pole formation, it is clear that other factors are involved in physically synthesizing new cell wall at these foci. This could be endogenous PonA1, but other factors are also likely involved in elongation. Identification of PonA1’s interacting partners would illuminate the composition of the elongation complex in mycobacteria and provide further understanding of how cell length and cell shape maintenance are controlled. For example, PonA1 and DivIVA might interact, even transiently, in the elongation complex. DivIVA is required for the rod-shape of mycobacteria [24] but has no enzymatic activity. It must coordinate cell growth with the enzymes that synthesize new cell wall [24]. PonA1, as a key factor that promotes cell elongation, may be one such factor that DivIVA helps position or facilitate its activity at the pole.

Even at hyper-physiological levels, PonA1 is properly recruited to the cell pole, as PonA1-RFP localizes at the pole before it degenerates into ectopic poles (S5 Fig). Furthermore, ectopic poles are not produced along the cell body. This suggests that the machinery that recruits PonA1 to the cell pole is not saturated even at increased levels of PonA1 nor is PonA1’s recruitment mechanism rate-limiting for elongation under physiological levels of PonA1. Additionally, the ectopic growth pole normally forms at one cell pole, although occasional cells with both cell ends sporting ectopic growth poles have been observed; however, these cells tend to have multiple septa (S4C Fig, white arrows). These data support a model of mycobacterial elongation where the cell poles are asymmetrically active, at least for a time[2,23,30].

Interestingly, the frequency of ectopic poles correlates with specific imbalanced PonA1 activity. The development of ectopic poles was highest with a PonA1 isoform that can crosslink PG but cannot polymerize glycan strands. The retention of transpeptidase activity in these mutants might lead to abnormal numbers of PG crosslinks. These data suggest that imbalanced crosslinking might contort local PG architecture, which may change the curvature of the membrane enough to misdirect elongation complexes to those foci (Fig 7B). Alternatively, PonA1 may nucleate elongation complexes at these contorted foci to then extend a functional growth pole. Although these ectopic poles elongate over time, they eventually block cell growth. These data suggest that PonA1’s coordination of polar growth must be tightly regulated to maintain linearity of the bacterial cell. This may be accomplished through proper protein-protein interactions and regulation of PonA1 activity.

### Phosphorylation regulates elongation rate

We found that cell growth is regulated through PonA1’s phosphorylation. Phosphorylation of PBPs is unusual, and only mycobacteria have so far been reported to phosphorylate their PBPs[6], suggesting that phosphorylation could regulate the activity of these proteins in mycobacteria. We found that the absence of PonA1’s phosphorylation increased the rate of single cell elongation (Fig 5), suggesting that PonA1’s phosphorylation normally dampens cell elongation in both *Msm* and *Mtb*. Cells of both species that harbor PonA1 alleles that cannot be phosphorylated (*Msm* T50A and *Mtb* T34A) are longer, suggesting that phosphorylation negatively regulates cell elongation (Fig 5). PonA1’s phosphorylation by PknB is consistent with PknB’s phosphorylation of other members of the PG biosynthetic pathway[39,40]. In fact, PknB regulates PG synthesis at each key point of the pathway: precursor synthesis (GlmU)[40], export (MviN)[39] and polymerization (PonA1). Interestingly, PknB’s phosphorylation of these three distinct factors is inhibitory, providing consistent regulation of PG biogenesis at multiple steps. It would be of interest to determine if PonA1 phosphorylation correlates with stages of the cell cycle; PonA1 may be phosphorylated as the cells begin the switch from elongation to septation, where cells need to slow down elongation. Alternatively, PonA1’s phosphorylation status may cycle with rounds of peptidoglycan synthesis during growth. Further work is required to test these models in addition to determining if PonA1’s phosphorylation impacts *Mtb* fitness during growth during infection.

How PonA1’s phosphorylation specifically regulates cell elongation is unknown, although our data suggest that it could act to regulate TG activity. In the absence of PonA1 phosphorylation, TG functionality could be hyperactive. This could lead to increased cell length observed with T50A cells (Fig 5) and correlates with the shortness of *Mtb* cells expressing TG- (Fig 1) or overexpressing TG- *Msm* cells (Figs 4 and S4). Additionally, when the T50A mutation is combined with TP-, *Msm* cells exhibit substantial defects in division (Fig 5), which could be a result of hyper-active glycan chain synthesis without corresponding crosslinking by PonA1’s *cis*-TP domain, altering the normal cellular response to PG synthesis. Differences in antibiotic susceptibility correlate between PonA1’s phosphorylation and enzymatic activity as well. In *Mtb*, PonA1_T34D_ and PonA1_TG-_ cells exhibit the same rifampicin tolerance (S10A Fig), whereas T34A and TP- have the same teicoplanin profile (Fig 6). In sum, these data suggest a model wherein PonA1’s phosphorylation regulates its TG activity (Fig 7A). How this could be achieved remains unclear, although it is possible that conformational changes occur upon phosphorylation. Alternatively, PonA1 phosphorylation may impact the stability of the elongation complex or of PonA1’s retention in the elongation complex, which could underlie the changes in cell length observed when cells express phosphorylation mutants. In any case, this is an unusual example of cytoplasmic regulation of periplasmic protein activity, though, given the number of phosphorylated proteins found in mycobacteria, this might be a common theme in these organisms.

### Altering PonA1 activity changes antibiotic susceptibility

Changes to PonA1 regulatory or enzymatic activity are required for robust growth during antibiotic treatment, suggesting that PonA1 mediates tolerance to drugs that target penicillin binding proteins or PG synthesis. We found markedly enhanced susceptibility of *Mtb* and *Msm* TP- cells to teicoplanin. Enhanced sensitivity of PonA1 TP- cells may reflect an increased cellular toxicity from imbalanced peptidoglycan synthesis. Recent work suggested that beta-lactams are particularly effective because they not only target TP domains of penicillin binding proteins but they also block crosslinking of nascent glycan strands, leading to an accumulation of non-crosslinked or improperly crosslinked strands that are then degraded, causing a futile cycle of synthesis and degradation that consumes resources[41]. Teicoplanin treated PonA1 TP- cells may experience this cellular toxicity. Because the TP- allele is likely still capable of transglycosylation, it can synthesize nascent glycan strands that will then be blocked from crosslinking when cells are treated with a crosslinking inhibitor. This could lead to an accumulation of uncrosslinked glycan strands and induce cellular toxicity, resulting in the increased teicoplanin sensitivity these cells exhibit. Because the T34A allele demonstrates a similar shifted MIC, it suggests that glycan strand synthesis is also altered in this mutant. Loss of phosphorylation may lead to enhanced TG activity, with a consequent imbalance of crosslinking of the new glycan chains when cells are treated with teicoplanin. Furthermore, the endogenous mycobacterial beta-lactamase is ineffective with drugs like teicoplanin, increasing their potential usefulness. These data suggest that transpeptidation inhibitors, such as teicoplanin, could be a fruitful avenue for TB drug development.

## Materials and Methods

### Bacterial strains and culture conditions

*M*. *smegmatis* mc^2^155 was cultured in Middlebrook 7H9 salts (Becto-Dickinson) supplemented with ADC (5 g/L albumin, 2 g/L dextrose, 3 g/L catalase), 0.25% glycerol, and 0.05% Tween-80 or plated on LB agar. *M*. *tuberculosis* H37Rv was cultured in Middlebrook 7H9 salts supplemented with OADC (oleic acid, albumin, dextrose, catalase [BD Biosciences, Franklin Lakes, NJ]), 0.25% glycerol, and 0.05% Tween-80 or plated on 7H10 agar. Selection was performed at 50 μg/ml hygromycin, 25 μg/ml kanamycin, 20 μg/ml zeocin or 20 μg/ml nourseothricin for both liquid and solid media. *E*. *coli* XL-1 Blue (Stratagene, Santa Clara, CA) or TOP10 (Invitrogen, Carlsbad, CA) were used for cloning and were cultured in LB broth or agar. Selection for *E*. *coli* occurred at 100 μg/ml hygromycin, 50 μg/ml kanamycin, 20 μg/ml zeocin or 50 μg/ml nourseothricin for both liquid and solid media. All strains were grown at 37°C. The generation of single copy PonA1 mutant strains is described further below. To generate multicopy PonA1 strains, alleles of *ponA1* were subcloned into an anhydrotetracycline inducible pMS2 derivative plasmid[42]. These plasmids were transformed into an *M*. *smegmatis* mc^2^155 strain with the pMC1s plasmid[42], encoding the *tetR* repressor, integrated at the L5 phage integration site.

### Recombinant DNA constructs

*M*. *smegmatis* PonA1 (MSMEG_6900) and *M*. *tuberculosis* PonA1 (Rv0050) catalytic mutants were generated using site directed mutagenesis and PCR stitching with the primers in S1 Table. *M*. *smegmatis* PonA1 truncation mutants were generated with primers listed in S1 Table. The double T50A,TP- Msm PonA1 construct was generated via PCR stitching using the appropriate primers in S1 Table. For all Mtb PonA1 constructs, the -426 start site was used. All constructs used in this study were cloned under the strong promoter pUV15 modified to contain TetR operator sites[42] (although no TetR is encoded in the cells’ genomes so expression of *ponA1* is constitutive). All PCR reactions were performed with KOD Xtreme Hot Start DNA polymerase (EMD Millipore, Billerica, MA). All *M*. *smegmatis* and *M*. *tuberculosis* PonA1 constructs listed above were cloned as translational fusions with either the FLAG epitope or monomeric red fluorescent protein (RFP). The FLAG epitope and RFP do not obstruct PonA1 function, as both tags recombineered onto the C-terminus of *ponA1* on the chromosome complement *Msm* survival.

### Deletion of ponA1 in *M*. *tuberculosis*

A *ponA1* (Rv0050) knockout construct was generated by PCR and subcloning. Because *ponA1* exists in an operon, the 5’ and 3’ 25 nucleotides of *ponA1* were preserved in the knockout construct. The 500 nucleotides upstream of *ponA1* were PCR amplified with a 3’ *NdeI* restriction endonuclease site. The 500 nucleotides downstream of *ponA1* were PCR amplified with a 5’ *PvuI* restriction endonuclease site. A hygromycin resistance cassette flanked by loxP sites was amplified with a 5’ *NdeI* site and a 3’ *PvuI* site. These three fragments were subcloned by digesting with *NdeI* and/or *PvuI*. The final ligated product was PCR amplified and used to replace *ponA1* in the *Mtb* H37Rv genome via recombineering[43]. Cells were plated on 7H10 plates with 50 μg/ml hygromycin. After incubation at 37°C for three weeks, colonies were inoculated into 2 ml of selective 7H9. Cell lysates were generated and screened by PCR for correct recombinants. One out of 11 colonies was positive for recombination; loss of *ponA1* from the genome was verified by PCR and whole genome sequencing.

### Ethics statement

The Institutional Animal Care and Use Committee of Harvard University approved and monitored all protocols, personnel, and animal use. The animal facilities are AAALAC accredited, and work was performed under the NIH Office of Laboratory Animal Welfare (OLAW) permit number A-3431-01.

### Mouse infections

Six week old wildtype female C57Bl6 mice (Jackson Laboratories, Bar Harbor, ME) were aerosol infected with low doses of *M*. *tuberculosis* H37Rv wildtype, H37Rv *ΔponA1*::*Hyg*, H37Rv *ΔponA1*::*Hyg* L5::*ponA1*_*wt*_, and H37Rv *ΔponA1*::*Hyg* L5::*ponA1*_TG-_. Strains were confirmed as PDIM positive by mass spectrometry prior to infection using established protocols[44]. Fifteen mice were infected per strain, and five mice were sacrificed at 1 day, 15 days, and 42 days post infection, respectively. To minimize suffering, animals were first put to sleep with isoflurane before sacrifice. Lungs and spleens were homogenized and serially diluted on selective 7H10 plates (for the genetically modified strains) or 7H10 plates lacking antibiotics (for H37Rv wildtype) for CFU enumeration.

### Allelic exchange in *M*. *smegmatis*

A marked copy of PonA1 was first integrated in the chromosome at the L5 phage integration site. The endogenous locus of PonA1 was then replaced with a different antibiotic cassette via recombineering. This dual marked strain was then transformed with an L5-integrating vector that is marked with a third antibiotic cassette. This second integration event replaces the original integrated allele, and selection for the third antibiotic recovers cells with the desired allele (Fig 1A). Exchange at the L5 site is imperfect and to confirm desired transformants, cells are patched onto plates containing the first L5 integrant marker or the second L5 integrant marker. Cells that are mono-resistant to the second L5 integrant antibiotic are counted as correct transformants; frequencies of correct transformation are calculated by comparison to wildtype *ponA1* control transformations.

### Bocillin labeling

FLAG-tagged isoforms of *M*. *smegmatis* PonA1 were produced in TOP10 *E*. *coli*. Untransformed TOP10 cells were used as a negative control. Cells were grown overnight in LB and back-diluted 1:100 into 10ml of LB. Once cells reached 0.5 OD_600_, they were pelleted. Cell pellets were washed once in 1ml of 1x PBS, resuspended in 1ml of 1x PBS, and sonicated twice for 30 seconds each. Bocillin-FL (Life Technologies) was added to the cell solutions at a final concentration of 15 μg/ml and allowed to label protein for 30 minutes at room temperature in the dark. The labeled cell solutions were then pelleted at 100,000 *g* for 20 minutes to concentrate membrane proteins. The pellets were resuspended in 100 μl of sample buffer containing βME. Proteins were resolved by SDS-PAGE using a 4–15% Tris-Glycine gel. Bocillin-labeled proteins were visualized by imaging with a Typhoon 9400 Variable Mode Imager (GE Healthcare).

### Timelapse microscopy and data analysis

For the data in Figs 5 and S8, vegetatively growing *M*. *smegmatis* cells were washed twice in 1X PBS with 0.1% Tween 20, stained with 50 μg/ml Alexa488 (Invitrogen), and filtered twice through a 10 μm filter to obtain single cells. Cells were inserted into custom microfluidic devices (see Reference [23]) and imaged at 37°C with constant flow of selective 7H9. Cells were imaged with a DeltaVision PersonalDV microscope using the 60x objective (Plan APO NA1.42) every 10 minutes for a minimum of 18 hours. Images were captured with a CoolSnap HQ2 camera (Photometric). Cell elongation and division events were annotated using ImageJ (National Institutes of Health) with the ObjectJ plugin (Norbert Vischer and Stelian Nastase, University of Amsterdam, http://simon.bio.uva.nl/objectj/index.html). Cell elongation was measured as new unstained cell wall material; cell division was defined as the first frame when physical invagination of the cell wall was visible. A custom Python script was used to analyze the annotations.

For the data in S4 Fig, *M*. *smegmatis* cells were imaged using a CellASIC microfluidic system (EMD Millipore, Billerica, MA). Vegetatively growing cells were cultured for four hours ± 100 ng/mL anhydrotetracycline (aTc) inducer to overexpress PonA1_TG—_RFP prior to growth in the CellASIC chambers. Cells were imaged every 15 minutes for 17 hours with constant flow of selective 7H9 ± 100 ng/mL aTc. Images were captured by a Nikon Eclipse TI microscope using the 60x objective, which is maintained at 37°C with an objective heater. Images were captured with a CoolSNAP SQ2 camera. The image montage in S4 Fig was compiled with FIJI[44].

### Light microscopy and image analysis

For imaging, cells were washed once in 1x PBS and resuspended in 1X PBS ± 2.5 μg/ml FM4-64Fx (Invitrogen) to visualize the plasma membrane. After resuspension, cells were incubated in the dark for 10 minutes prior to imaging. For *M*. *tuberculosis*, cells were first fixed overnight in 1% formalin in the biosafety level 3 facility before removal and consequent imaging. Cells were imaged on a Nikon TE-200E microscope using the 100x (NA1.40) objective. Images were captured with an Orca-II ER cooled CCD camera (Hamamatsu, Japan). Exposure and image acquisition were controlled with Metamorph Software (Molecular Devices). Final images were prepared in Adobe Photoshop CS3. To quantitate cell length, the fluorescent images of cells stained with FM4-64Fx were used. Cell length was calculated using ImageJ software (National Institutes of Health) and converted to microns using the appropriate pixel to micron conversion. Length was measured from cell pole to the opposite cell pole or from cell pole to septum, if present. Cell synchronization is not yet possible for mycobacteria; hence, cell lengths were measured for unsynchronized populations. To quantitate the frequency of multi-poled cells, cells were both imaged and scored blind to eliminate potential bias. The figure shows data from five combined experiments. 2652 cells were imaged and counted for PonA1_wt_, 2910 cells for PonA1_TG-_ cells, 2054 for PonA1_TP-_ cells, and 2379 for PonA1_TG-TP-_ cells. The control represents data collected from uninduced cells. Overexpression of PonA1 was induced with 100 ng/mL aTc.

### *In vitro* kinase assays and detection of PonA1’s phosphorylation site

N-terminally his-MBP-tagged kinase domains of the nine canonical serine-threonine protein kinases from *Mtb* were expressed and purified from *E*. *coli* using a nickel column and then an S75 size exclusion column, in kinase buffer (50 mM Tris pH7.5, 150 mM NaCl, 20% glycerol). His-MBP-PonA1 (cytoplasmic domain) was expressed and purified from *E*. *coli* using Ni-NTA beads (Novagen) according to the protocol recommended by the manufacturer. Protein levels were measured by A280, and kinase reactions were started by mixing 1 μg of kinase, 10 μg of his-MBP-PonA1(cyto), 1mM MnCl_2_ and 1mM ATP. Reactions were incubated at room temperature for 1 hour and stopped by addition of Laemmli buffer. Samples were heated, separated by SDS-PAGE, and detected by western blot using α-phospho-threonine antibody (Cell Signaling Technology). Kinase reactions for mass spectrometry were performed the same way, using only his-MBP-PknB and his-MBP-PonA1(cyto). The his-MBP-PonA1 protein band was cut out of the polyacrylamide gel and in-gel trypsin digested. Samples were analyzed by liquid chromatography (LC)/MS/MS with an Agilent 6520 Accurate-Mass Quadrupole Time-of-Flight instrument. Peptides were separated on a POROSHELL 300SB-C18 (2.1 × 75 mm, 5 μm) at a 0.5 mL/min flow rate, by using a linear gradient of increasing acetonitrile in water. Spectrum Mill software (Agilent) was used to identify peptides.

### Antibiotic treatment and determination of bacterial cell fitness

The antibacterial effects of teicoplanin (Sigma Aldrich, St. Louis, MO) and moenomycin A (Sigma Aldrich, St. Louis, MO) were determined by culturing exponentially growing *M*. *tuberculosis* or *M*. *smegmatis* cells in complete 7H9 in non-treated 96 well plates (Genesee Scientific, San Diego, CA) in the presence of serially diluted drug, in duplicate. Plates also contained duplicate untreated wells as controls. *M*. *tuberculosis* was grown to OD_600_ of 0.5 before culturing ± drug at a calculated starting OD_600_ of 0.006 for six days at 37°C with shaking. *M*. *smegmatis* was grown to OD_600_ of 0.5 before culturing ± drug at a calculated starting OD_600_ of 0.05 for 16 hours at 37°C with shaking. Bacterial growth was evaluated by adding 0.002% resazurin (Alamar Blue, Sigma Aldrich, St. Louis, MO) to each well. *M*. *tuberculosis* plates were incubated with resazurin for 18–24 hours. *M*. *smegmatis* plates were incubated with resazurin for 3–5 hours. Wells were scored as blue or purple indicating metabolically inactive cells and pink as metabolically active cells. The inhibitory effects of meropenem (Sigma Aldrich, St. Louis, MO) were determined by growing *M*. *smegmatis* to an OD_600_ of 0.5 before culturing ± drug at a calculated starting OD_600_ of 0.1. Cells were grown in complete 7H9 in 96 well honeycomb plates (Growth Curves USA, Piscataway, NJ) ± drug, in triplicate, at 37°C with shaking in a Bioscreen growth curve machine (Growth Curves USA, Piscataway, NJ). Optical density was measured by absorbance at 600nm every 30 minutes for 48 hours. The inhibitory effects of rifampicin (Sigma Aldrich, St. Louis, MO) were determined by growing *M*. *tuberculosis* to an OD_600_ of 0.5 before culturing ± drug at a calculated starting OD_600_ of 0.1. Cells were grown in complete 7H9 in 24 well untreated plates ± drug, in triplicate, at 37°C with shaking. Optical density was measured after six days, and growth was normalized to untreated control wells.

### Optical density measurements

Population growth curves for *M*. *smegmatis* strains were performed in 96 well honeycomb plates (Growth Curves USA, Piscataway, NJ), in triplicate, at 37°C with shaking in a Bioscreen growth curve machine (Growth Curves USA, Piscataway, NJ). Cells were grown to an OD_600_ of 0.5 before beginning the growth curve at a calculated starting OD_600_ of 0.1. Cells were grown in complete 7H9 with the appropriate antibiotic selection. Optical density was measured by absorbance at 600nm every 30 minutes for 48 hours. Error bars are often too small to see. Population growth curves for *M*. *tuberculosis* strains were performed in 30 ml inkwells (Corning Life Sciences, Corning, NY), in triplicate, at 37°C with shaking. Cells were grown to an OD_600_ of 0.5 before beginning the growth curve at a calculated starting OD_600_ of 0.1. Cells were grown in complete 7H9 with the appropriate antibiotic selection. Optical density was measured daily by absorbance at 600nm.

### Immunoblotting

Cells were pelleted, washed once in 1X PBS, and lysed by bead-beating. 6X Laemmli buffer was added to the whole cell lysate and then boiled for 10 minutes. Proteins were separated on a 12–14% Tris-Glycine SDS PAGE gels (BioRad, Hercules, CA), transferred to PVDF membrane (Pall Corp, Pensacola, FL), incubated with anti-FLAG antibodies (Sigma Aldrich, St. Louis, MO) at 1:15,000 dilution, washed thrice in 1X PBST, and incubated with a 1:1000 dilution of a secondary antibody conjugated to HRP (Pierce, Rockford, IL). Membranes were incubated with SuperSignal chemiluminescent reagent (Thermo Fisher Scientific, Rockford, IL) for 5 minutes in the dark and imaged on a FluorChem8900 (AlphaInnotech, Santa Clara, CA).

### Data representation and statistical analysis

Prism 6.0 software (GraphPad Software, La Jolla, CA) was used to graph and analyze numerical data. Statistical tests in the Prism software were used to calculate significance of measurements as described in the figure legends. The doubling times in S2 Fig and S9 Fig were generated by fitting an exponential growth function (in Prism) to all datapoints between 5 and 13 hours of OD_600_ measurements.

## Supporting Information

S1 Fig Changes to PonA1 function modestly impacts *Mtb* morphology. **(A)** Cells that no longer express PonA1 or that express PonA1 catalytic or regulatory mutants exhibit no gross morphological changes. Scale bar, 2 μm. **(B)** Population doubling is not severely impacted by loss of PonA1 when cells are grown in standard laboratory conditions. **(C)** Expression of catalytic mutants of PonA1 does not significantly impact population doubling rates, suggesting that the defect observed during infection is not due to changes in growth rate. **(D)** Similarly, changes to PonA1’s regulatory activity do not significantly change population doubling rates.
(TIF)

S2 Fig Normal cell growth requires a fully functional PonA1. **(A)** A panel of PonA1 mutants was constructed to investigate the cellular role of PonA1. Catalytic mutations in the TG or TP domain replace the active site serine with an alanine to abolish enzymatic activity (red X). The phosphorylation mutations either remove the phosphorylation site (yellow bar) by replacing the phosphorylated threonine with an alanine (lack of yellow bar) or attempt to mimic the phosphorylation with a threonine to aspartic acid mutation (dark red bar). The truncation mutations were deletion of the majority of the cytoplasmic tail (*Msm* PonA1_95-827_) or of the TP domain (*Msm* PonA1_1-360_). **(B)** FLAG immunoblotting demonstrates that *E*. *coli* cells express *Msm* PonA1-FLAG. Lane 1, negative control (wildtype *E*. *coli*); lane 2, *Msm* PonA1_wt_-FLAG, lane 3, *Msm* PonA1_TP—_FLAG, lane 4, *Msm* PonA1_TG—_FLAG, lane 5, *Msm* PonA1_TG-TP—_FLAG. **(C)** Expression of PonA1_TP—_FLAG complements bacterial growth, although population doubling rates are slightly dampened. **(D)** During exponential growth, the TP- cells have an average doubling time of 4.21 hours, whereas isogenic wildtype doubles on average every 3.40 hours (p-value < 0.0001 by the unpaired two-tailed t-test). **(E)** The PonA1_TP—_FLAG isoform is stable, suggesting the phenotype of short cell length is due to lack of PonA1’s PG crosslinking. **(F)** Expression of an allele that encodes only PonA1’s TG domain (PonA1_1-360_-FLAG) complements bacterial survival, although it dampens population doubling rates. **(G)** During exponential growth, PonA1_1-360_ cells double on average every 4.23 hours, whereas isogenic wildtype doubled every 3.45 hours in this experiment (p-value < 0.0001 by the unpaired two-tailed t-test). **(H)** The PonA1_1-360_-FLAG protein is stable, suggesting the cell shape changes observed are due to changes in PonA1 function because of the truncated allele and not due to an unstable protein isoform.
(TIF)

S3 Fig Mass spectrometric quantitation of pthiocerol dimycocerosate (PDIM) for strains used for mouse infections. Total cell wall lipids from *M*. *tuberculosis* in mid-log phase growth were extracted with chloroform:methanol and quantitated using established liquid chromatography-mass spectrometry protocols[45]. Individual PDIM A and PDIM B species were identified based on characteristic retention times and highly accurate mass matching (NH4+ adducts).
(TIF)

S4 Fig Overproduction of PonA1 mutants changes *Msm* cell shape. **(A)** Cells that overexpress different catalytic variants of PonA1 exhibit cell shape changes, including ectopic polar growth, bulging poles, and altered cell length. Cells were imaged six hours of induction. Scale bar, 2 μm. **(B)** Quantitation of cell length of cells in (A). A TG- allele of PonA1 negatively impacts cell length more than other catalytic variants, perhaps because these cells also produce the highest frequency of ectopic poles. Cells that do not exhibit an ectopic pole are shorter than wildtype, however, which may suggest a role for balanced PG synthesis in productive activity of the elongation complex (control: 237 cells; wildtype: 226 cells; TG-: 244 cells; TP-: 163 cells; TG-TP-: 234 cells; representative data. Significance was assessed by the Kolmogorov-Smirnov test. PonA1_wt_ compared to PonA1_TG-_ approximate p-value < 0.0001; PonA1_wt_ compared to PonA1_TP-_ approximate p-value < 0.0001; PonA1_wt_ compared to PonA1_TG-TP-_ approximate p-value < 0.0001). **(C)** Overexpression of PonA1 leads to ectopic poles usually at one pole; rare cells are observed with both poles having formed ectopic poles. However, these cells usually exhibit multiple septa (white arrows), indicating these cells are not truly uni-cellular and are not an accurate reflection of ‘symmetrically’ active growth poles. **(D)** Endogenous PonA1 tagged with RFP on the chromosome localizes to the cell pole and mid-cell in *M*. *smegmatis*.
(TIF)

S5 Fig PonA1 is an early polar localizing factor that drives polar growth. **(A)** *Msm* cells that encode an overexpression vector for the TG- allele of PonA1-RFP were grown ± inducer to overproduce PonA1_TG—_RFP for four hours. The cells were then imaged for 17 hours ± inducer in the CellASIC microfluidic system to visualize cell growth. Cells that overexpress PonA1_TG—_RFP exhibit slow population growth as previously observed. PonA1 localizes to the pole prior to budding of the ectopic pole (follow cell with white arrow), suggesting that PonA1 is an early localizing factor at the growth tip and drives growth of the pole or ectopic pole upon PonA1 overproduction. Scale bar, 2 μm. **(B)** PonA1_TG—_RFP cells grown without inducer exhibit normal morphology in the CellASIC microfluidic system and grow robustly. Scale bar, 2 μm.
(TIF)

S6 Fig Mycobacterial PonA1 encodes a phosphorylated cytoplasmic domain. **(A)** We used an H37Rv PonA1 (*rv0050*) construct with a start site 426 nucleotides upstream of the annotated start. This start site generates a protein with a predicted transmembrane pass, as expected for PBPs, and captures the translational start site. Alignment of the start site for *Msm* PonA1 (MSMEG_6900) with the *Mtb* genes shifts the start site by 126 nucleotides upstream. **(B)** The -426 *Mtb* PonA1 and -126 *Msm* PonA1 protein align well with the CDC1551 sequence for PonA (proteins were aligned with ClustalO on the EBI server). These proteins contain a phosphorylated threonine (H37Rv PonA1 T34A; *Msm* PonA1 T50A, yellow box). **(C)** Truncation of PonA1’s cytoplasmic tail or alteration of its phosphorylation site do not alter protein stability, suggesting the observed phenotypes are not due to aberrant protein production or folding.
(TIF)

S7 Fig *M*. *tuberculosis* PonA1 complements *M*. *smegmatis* survival. **(A)** *Msm* cells that express *Mtb* -426 PonA1 show no morphological differences. Scale bar, 2 μm. **(B)** The *Msm* cells that only express *Mtb* -426 PonA1 also double at rates identical to isogenic wildtype, suggesting that -426 PonA1 fully complements growth of *Msm* that lacks endogenous PonA1. **(C)** The *Mtb* PonA1 allele is produced at similar levels to *Msm* PonA1 (the nonspecific band demonstrates lane 2 has less protein loaded). **(D)** Mass spectrometric analysis confirms that PknB phosphorylates *Mtb* MBP-PonA1_cyto_ (with the -426 start site) *in vitro*.
(TIF)

S8 Fig PonA1 phosphorylation regulates single cell elongation rates. **(A)** *Msm* cells that express phosphorylation mutants (T50A and T50D) or a truncation of the cytoplasmic tail of PonA1 (*Δ*cyto) exhibit similar population doubling rates to isogenic wildtype or wildtype *Msm*. **(B)** To investigate the impact of PonA1’s phosphorylation on cell elongation and division, cells were stained with a green fluorescent dye that binds to the cell surface. After staining, single cells were imaged in custom microfluidic devices, and new cell wall elongation and division events were measured. **(C)** The increase in single cell elongation rate of cells that express PonA1_T50A_ correlates with an increased length of single cells at division, as expected. (PonA1_wt_ compared with PonA1_T50A_ approximate p-value < 0.0001 by the Kolmogorov-Smirnov test. PonA1_wt_ compared with PonA1_Δcyto_ approximate p-value < 0.0001 by the Kolmogorov-Smirnov test). **(D)** Expression of T50A or *Δ*cyto PonA1 do not impact single cell generation times. This suggests that the observed increased cell length is due to faster single cell elongation rates alone and is not impacted by altered septation timing. Significance was assessed by the Kolmogorov-Smirnov test, and neither mutant population was statistically different than PonA1_wt_. **(E)** Cell elongation still occurs predominantly from the old pole in the absence of PonA1’s phosphorylation or cytoplasmic tail. (PonA1_wt_ compared with PonA1_T50A_ approximate p-value < 0.0001 by the Kolmogorov-Smirnov test. PonA1_wt_ compared with PonA1_Δcyto_ approximate p-value < 0.0001 by the Kolmogorov-Smirnov test). **(F)** The new pole exhibits mild increased elongation in cells that express T50A or *Δ*cyto PonA1 compared to wildtype. (PonA1_wt_ compared with PonA1_T50A_ approximate p-value = 0.0036 by the Kolmogorov-Smirnov test. PonA1_wt_ compared with PonA1_Δcyto_ approximate p-value = 0.0261 by the Kolmogorov-Smirnov test). Together with (E), these data suggest that loss of PonA1’s phosphorylation does not impact subcellular distribution of elongation complexes or PonA1’s localization within the elongation complex itself, since the majority of cell elongation still occurs at the old pole.
(TIF)

S9 Fig Inactivating PonA1’s phosphorylation site and TP active site together impacts cell growth. **(A)** Cells that express a T50A,TP- allele of PonA1 exhibit slower population doubling time as compared to the single point mutants or isogenic wildtype, suggesting that PonA1’s phosphorylation may regulate TG activity to promote normal cell elongation and division. **(B)** T50A cells double on average every 3.52 hours, TP- cells double every 4.21 hours (p-value < 0.0001 by the unpaired t-test compared to isogenic wildtype), and T50A,TP- cells double every 4.92 hours (p-value < 0.0001 by the unpaired t-test compared to isogenic wildtype; p-value = 0.0008 compared to TP- cells; p-value < 0.0001 by the unpaired t-test compared to T50A cells), whereas isogenic wildtype doubles every 3.40 hours. **(C)** The T50A,TP- allele is translated into a stable protein.
(TIF)

S10 Fig Antibiotic tolerance shifts when PonA1’s activity changes. **(A)** PonA1 single nucleotide polymorphisms identified in clinical isolates have previously been shown to change the tolerance of *M*. *tuberculosis* to rifampicin, a frontline tuberculosis therapy. We measured the impact of PonA1 catalytic and regulatory mutants on cell fitness during rifampicin treatment, and found that PonA1_TG-_ and PonA1_T34D_ mutants have 5- and 4-fold increased tolerance to rifampicin compared to isogenic wildtype. Other catalytic (TP-, TG-TP-) or regulatory (T34A) mutations do not alter rifampicin susceptibility. These data suggest that PonA1’s phosphorylation may regulate PonA1’s TG activity and that alterations to PonA1 function impact rifampicin tolerance. (Statistical significance was assessed by one-way analysis of variance with Bonferroni’s multiple comparison test, and multiplicity adjusted p-values are reported. PonA1_wt_ compared to PonA1_TG-_ p-value < 0.0001; PonA1_wt_ compared to PonA1_TG-TP-_ p-value = 0.0295; PonA1_wt_ compared to PonA1_T34D_ p-value < 0.0001). **(B)** Changes to PonA1 activity also influence cell fitness in the presence of TP domain inhibitors, including those that target both d,d- and l,d-transpeptidases (meropenem). The expression of T50A PonA1 impacts *M*. *smegmatis* cell fitness during meropenem treatment, corroborating the importance of normal PonA1 regulatory activity in the maintenance of cell fitness during antibiotic pressure. **(C)** The TG inhibitor moenomycin exhibits modest efficacy against *Msm* and *Mtb*.
(TIF)

S11 Fig Normal PonA1 activity is required for cell fitness during stress. **(A)** Cells that lack PonA1’s TP activity are less fit during incubation with SDS as compared to isogenic wildtype cells. This suggests that loss of PonA1’s PG crosslinking impinges on cell wall integrity. **(B)** Cells that express a PonA1 T50A allele also exhibit a modest defect in population doubling in the presence of SDS, suggesting changes to PonA1’s phosphorylation status also impact cell wall integrity. **(C)** Incubating isogenic wildtype *Msm* cells in the presence of D-amino acids, which may be incorporated into the cell wall, shows that PonA1’s PG crosslinking may be important for properly incorporating these non-canonical amino acids into the cell wall. **(D)** Cells that lack PonA1’s TP activity exhibit less robust population growth in the presence of D-Met. **(E)** Cells that express a TP- PonA1 exhibit cell shape defects when in deep stationary phase (cultured for four days). Cells become wider, shorter and rounder than isogenic wildtype, suggesting that PonA1’s crosslinking is important for PG integrity during stress.
(TIF)

S1 Table Primers used to generate recombinant DNA constructs. (XLSX)

S1 Video PonA1 localizes to the pole before it degenerates into ectopic poles. (ZIP)

S2 Video Cells that do not overexpress PonA1 do not form ectopic poles. (ZIP)
