# Supplementary figures and images for: Phosphorylation of the Peptidoglycan Synthase PonA1 Governs the Rate of Polar Elongation in Mycobacteria
Source: PLoS Pathog. 2015 Jun 26;11(6):e1005010. doi: 10.1371/journal.ppat.1005010 (PMC4483258; doi:10.1371/journal.ppat.1005010)

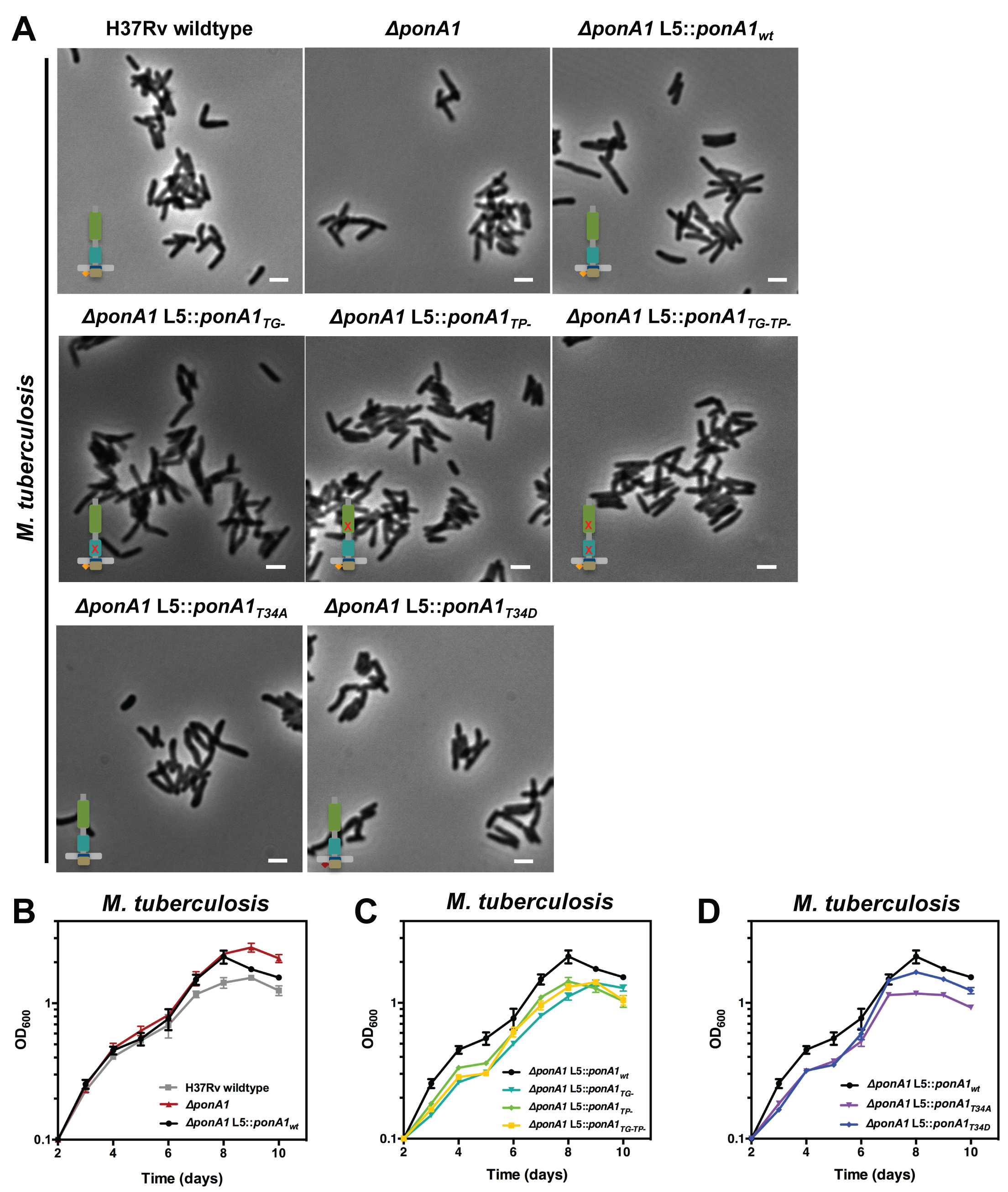

Supplement: S1 Fig — (A) Cells that no longer express PonA1 or that express PonA1 catalytic or regulatory mutants exhibit no gross morphological changes. Scale bar, 2 μm. (B) Population doubling is not severely impacted by loss of PonA1 when cells are grown in standard laboratory conditions. (C) Expression of catalytic mutants of PonA1 does not significantly impact population doubling rates, suggesting that the defect observed during infection is not due to changes in growth rate. (D) Similarly, changes to PonA1’s regulatory activity do not significantly change population doubling rates. (TIF) [file ppat.1005010.s001.tif]

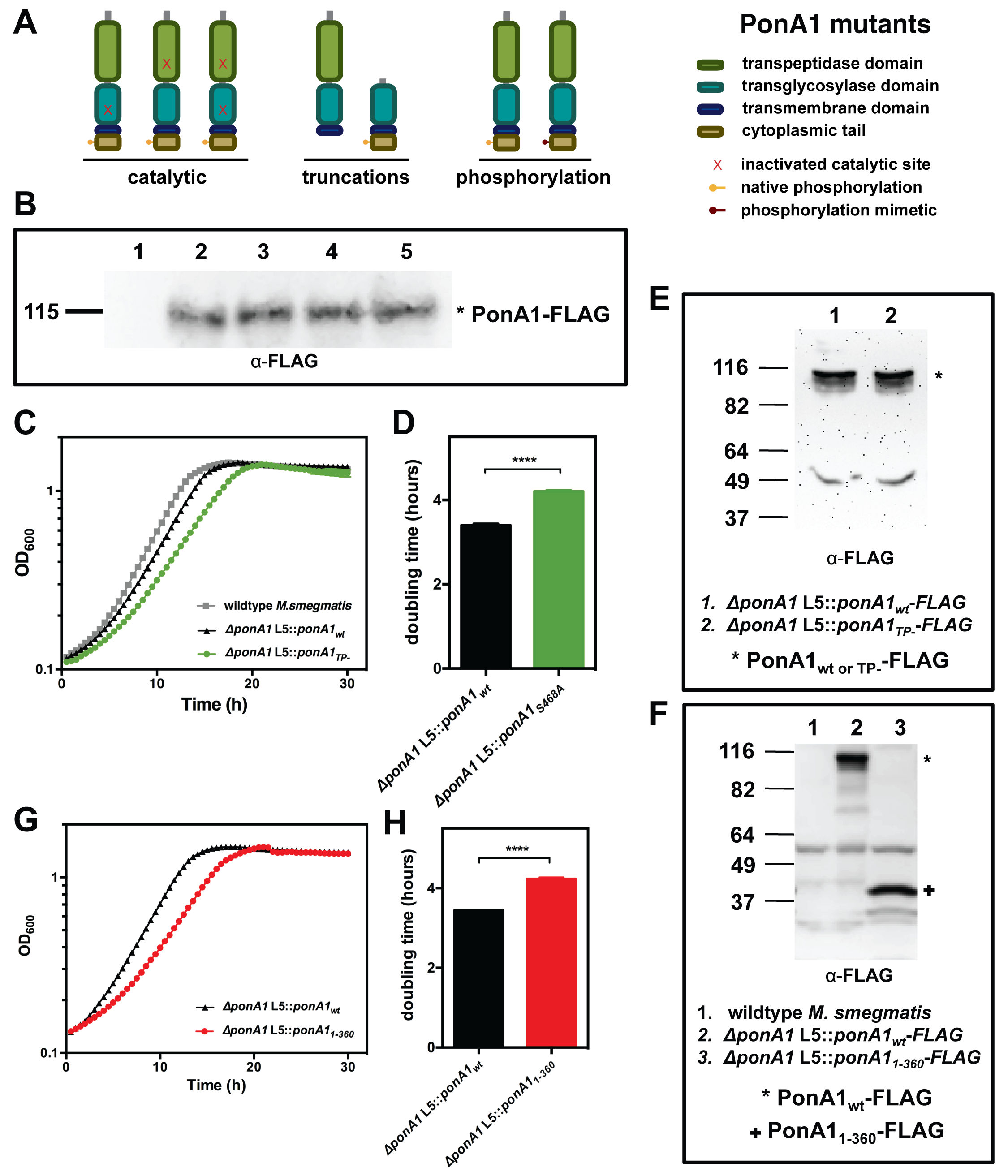

Supplement: S2 Fig — (A) A panel of PonA1 mutants was constructed to investigate the cellular role of PonA1. Catalytic mutations in the TG or TP domain replace the active site serine with an alanine to abolish enzymatic activity (red X). The phosphorylation mutations either remove the phosphorylation site (yellow bar) by replacing the phosphorylated threonine with an alanine (lack of yellow bar) or attempt to mimic the phosphorylation with a threonine to aspartic acid mutation (dark red bar). The truncation mutations were deletion of the majority of the cytoplasmic tail (Msm PonA195-827) or of the TP domain (Msm PonA11-360). (B) FLAG immunoblotting demonstrates that E. coli cells express Msm PonA1-FLAG. Lane 1, negative control (wildtype E. coli); lane 2, Msm PonA1wt-FLAG, lane 3, Msm PonA1TP—FLAG, lane 4, Msm PonA1TG—FLAG, lane 5, Msm PonA1TG-TP—FLAG. (C) Expression of PonA1TP—FLAG complements bacterial growth, although population doubling rates are slightly dampened. (D) During exponential growth, the TP- cells have an average doubling time of 4.21 hours, whereas isogenic wildtype doubles on average every 3.40 hours (p-value < 0.0001 by the unpaired two-tailed t-test). (E) The PonA1TP—FLAG isoform is stable, suggesting the phenotype of short cell length is due to lack of PonA1’s PG crosslinking. (F) Expression of an allele that encodes only PonA1’s TG domain (PonA11-360-FLAG) complements bacterial survival, although it dampens population doubling rates. (G) During exponential growth, PonA11-360 cells double on average every 4.23 hours, whereas isogenic wildtype doubled every 3.45 hours in this experiment (p-value < 0.0001 by the unpaired two-tailed t-test). (H) The PonA11-360-FLAG protein is stable, suggesting the cell shape changes observed are due to changes in PonA1 function because of the truncated allele and not due to an unstable protein isoform. (TIF) [file ppat.1005010.s002.tif]

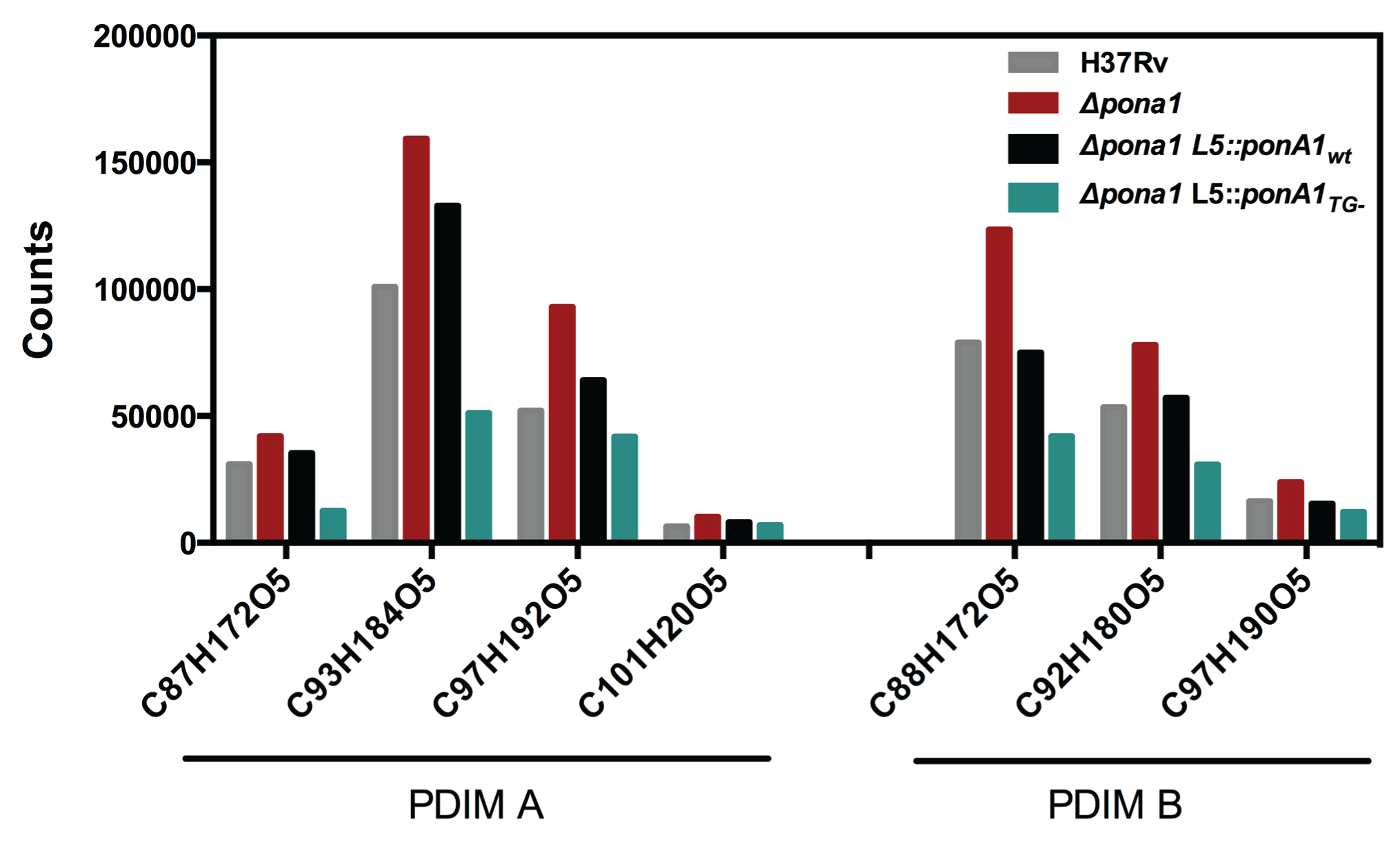

Supplement: S3 Fig — Total cell wall lipids from M. tuberculosis in mid-log phase growth were extracted with chloroform:methanol and quantitated using established liquid chromatography-mass spectrometry protocols[45]. Individual PDIM A and PDIM B species were identified based on characteristic retention times and highly accurate mass matching (NH4+ adducts). (TIF) [file ppat.1005010.s003.tif]

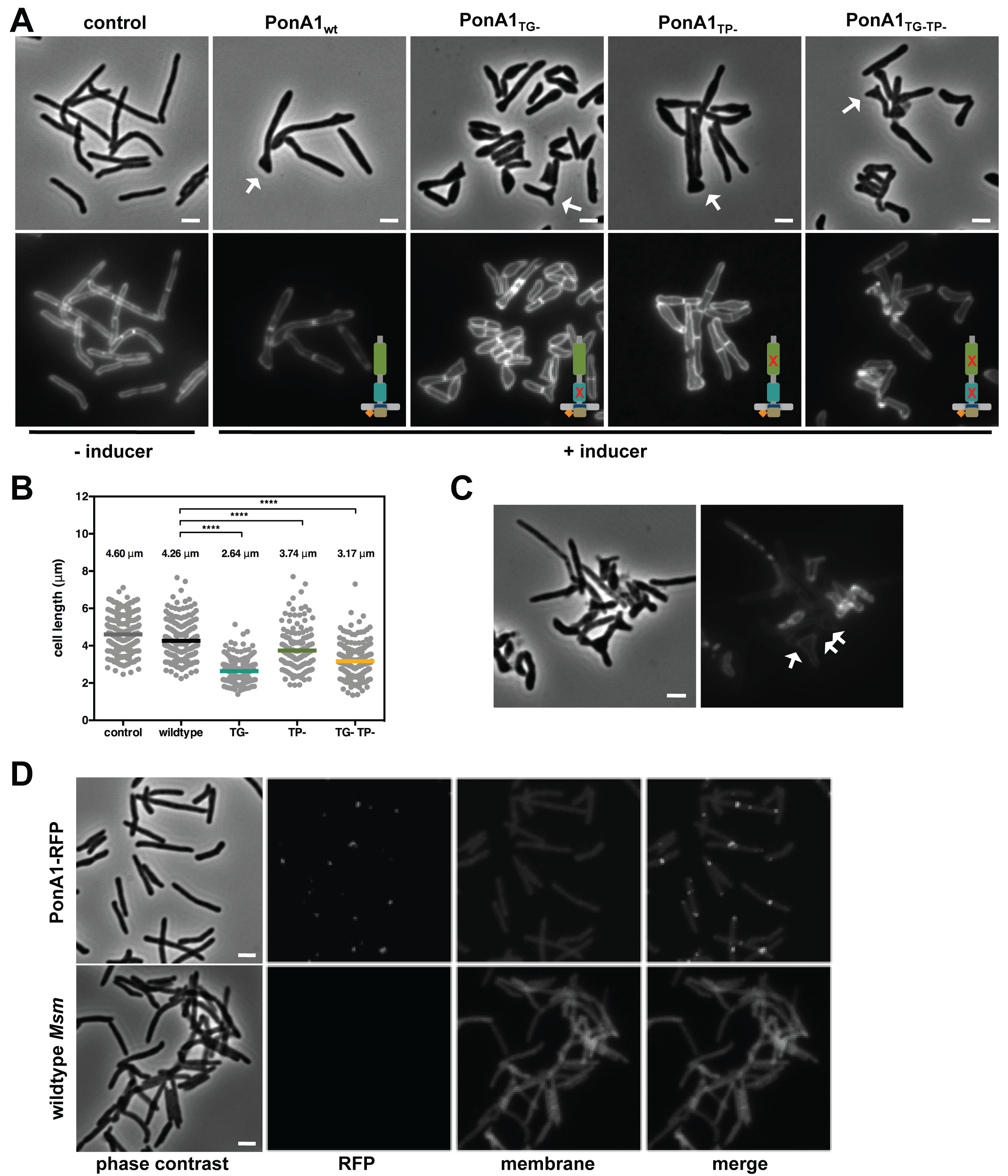

Supplement: S4 Fig — (A) Cells that overexpress different catalytic variants of PonA1 exhibit cell shape changes, including ectopic polar growth, bulging poles, and altered cell length. Cells were imaged six hours of induction. Scale bar, 2 μm. (B) Quantitation of cell length of cells in (A). A TG- allele of PonA1 negatively impacts cell length more than other catalytic variants, perhaps because these cells also produce the highest frequency of ectopic poles. Cells that do not exhibit an ectopic pole are shorter than wildtype, however, which may suggest a role for balanced PG synthesis in productive activity of the elongation complex (control: 237 cells; wildtype: 226 cells; TG-: 244 cells; TP-: 163 cells; TG-TP-: 234 cells; representative data. Significance was assessed by the Kolmogorov-Smirnov test. PonA1wt compared to PonA1TG- approximate p-value < 0.0001; PonA1wt compared to PonA1TP- approximate p-value < 0.0001; PonA1wt compared to PonA1TG-TP- approximate p-value < 0.0001). (C) Overexpression of PonA1 leads to ectopic poles usually at one pole; rare cells are observed with both poles having formed ectopic poles. However, these cells usually exhibit multiple septa (white arrows), indicating these cells are not truly uni-cellular and are not an accurate reflection of ‘symmetrically’ active growth poles. (D) Endogenous PonA1 tagged with RFP on the chromosome localizes to the cell pole and mid-cell in M. smegmatis. (TIF) [file ppat.1005010.s004.tif]

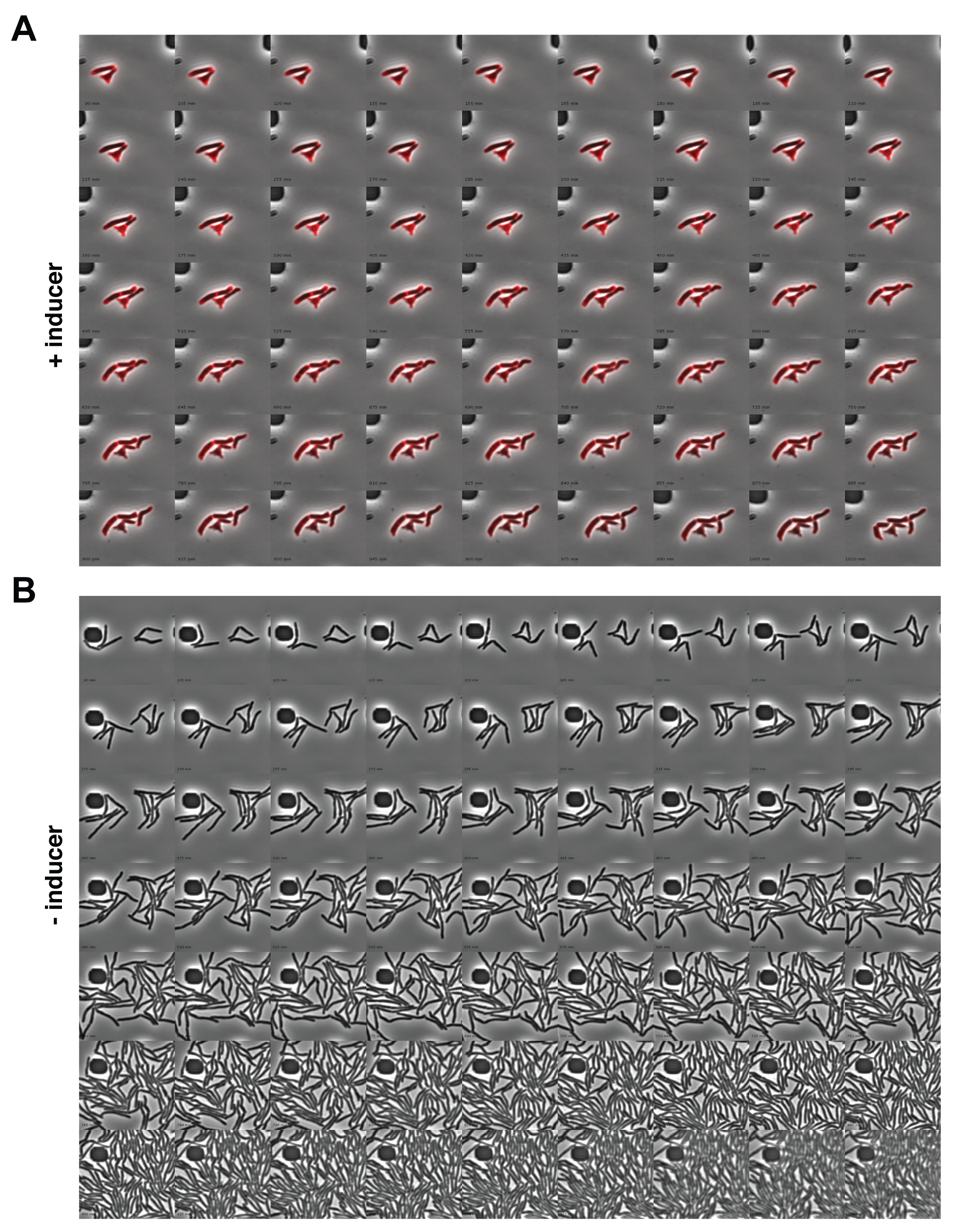

Supplement: S5 Fig — (A) Msm cells that encode an overexpression vector for the TG- allele of PonA1-RFP were grown ± inducer to overproduce PonA1TG—RFP for four hours. The cells were then imaged for 17 hours ± inducer in the CellASIC microfluidic system to visualize cell growth. Cells that overexpress PonA1TG—RFP exhibit slow population growth as previously observed. PonA1 localizes to the pole prior to budding of the ectopic pole (follow cell with white arrow), suggesting that PonA1 is an early localizing factor at the growth tip and drives growth of the pole or ectopic pole upon PonA1 overproduction. Scale bar, 2 μm. (B) PonA1TG—RFP cells grown without inducer exhibit normal morphology in the CellASIC microfluidic system and grow robustly. Scale bar, 2 μm. (TIF) [file ppat.1005010.s005.tif]

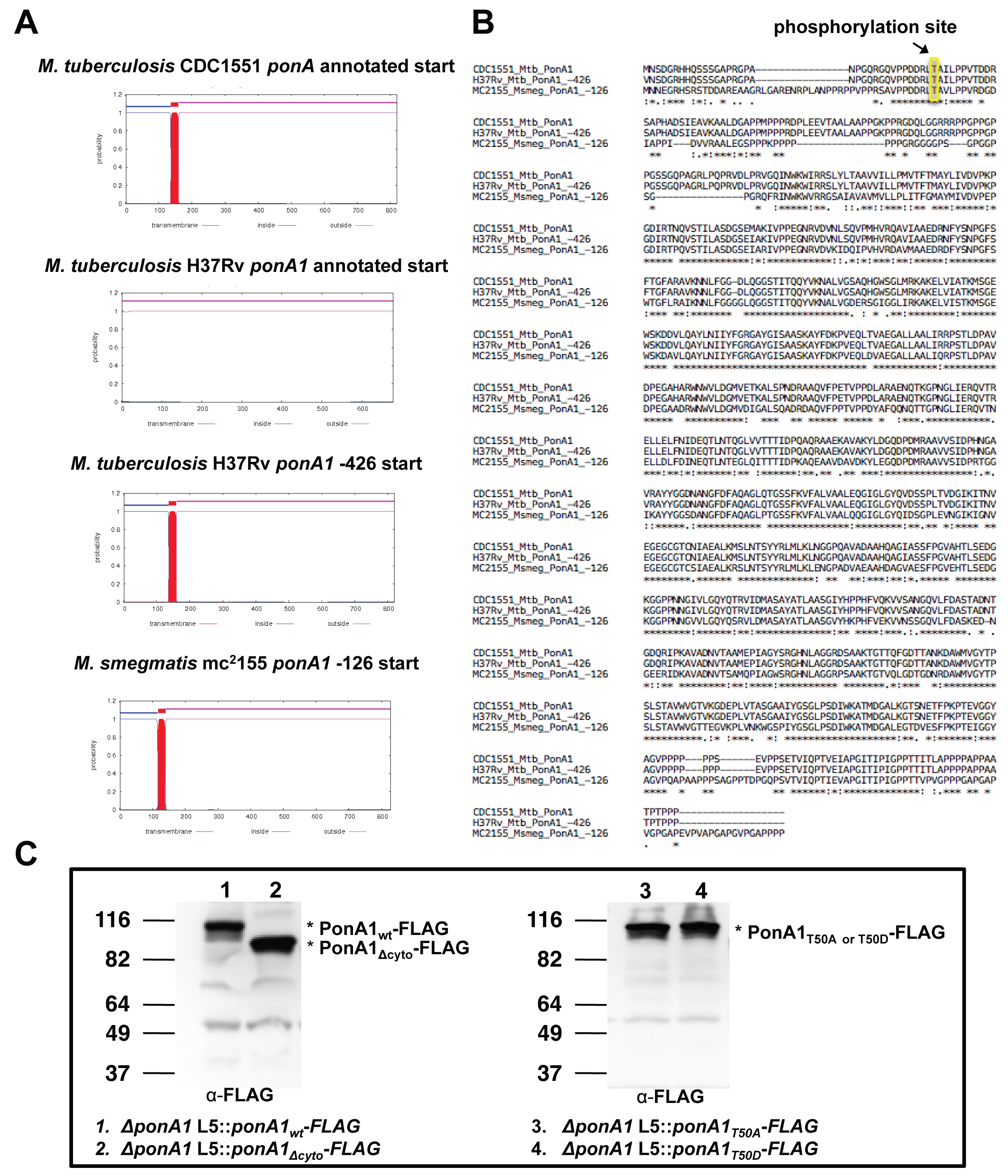

Supplement: S6 Fig — (A) We used an H37Rv PonA1 (rv0050) construct with a start site 426 nucleotides upstream of the annotated start. This start site generates a protein with a predicted transmembrane pass, as expected for PBPs, and captures the translational start site. Alignment of the start site for Msm PonA1 (MSMEG_6900) with the Mtb genes shifts the start site by 126 nucleotides upstream. (B) The -426 Mtb PonA1 and -126 Msm PonA1 protein align well with the CDC1551 sequence for PonA (proteins were aligned with ClustalO on the EBI server). These proteins contain a phosphorylated threonine (H37Rv PonA1 T34A; Msm PonA1 T50A, yellow box). (C) Truncation of PonA1’s cytoplasmic tail or alteration of its phosphorylation site do not alter protein stability, suggesting the observed phenotypes are not due to aberrant protein production or folding. (TIF) [file ppat.1005010.s006.tif]

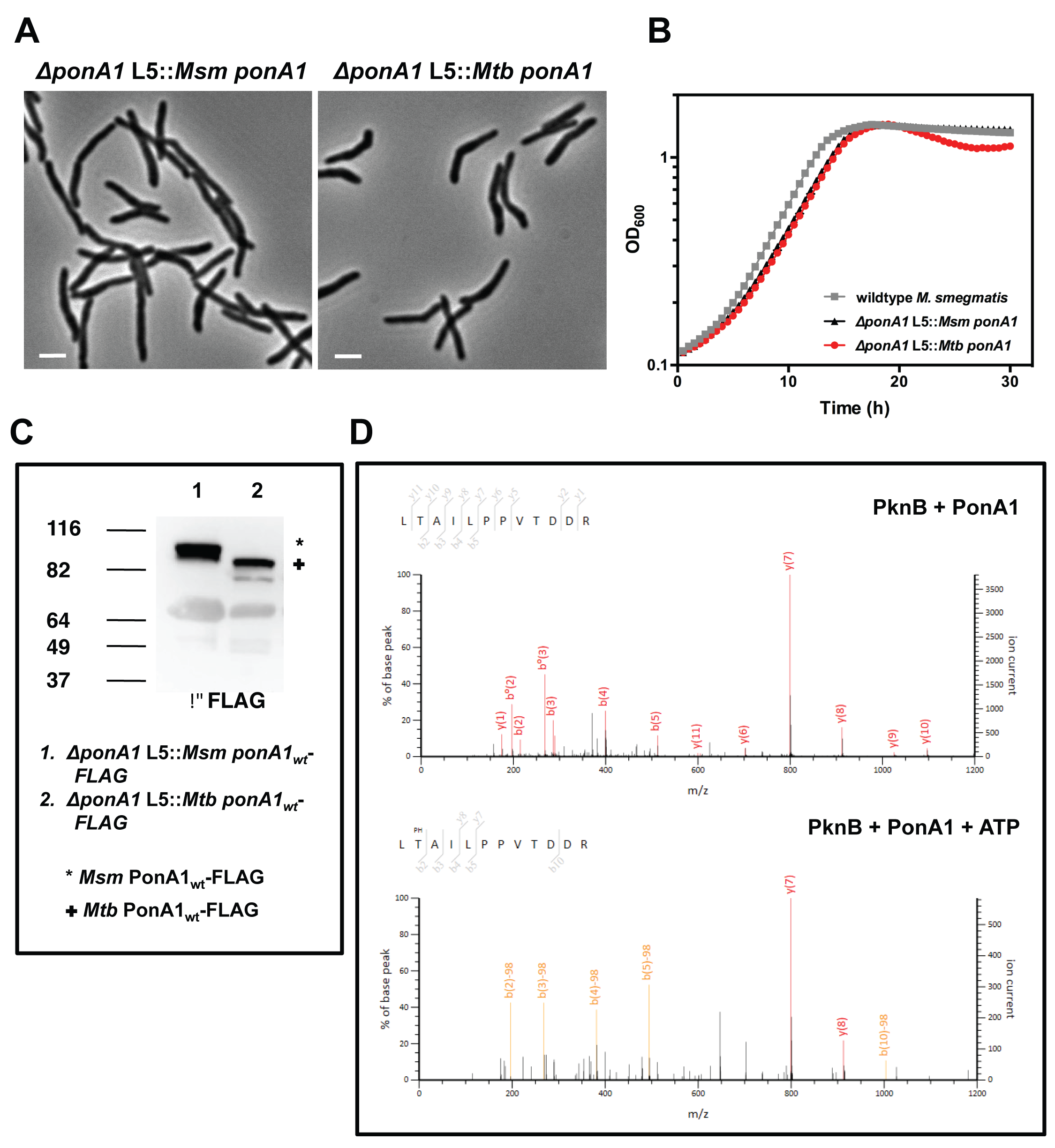

Supplement: S7 Fig — (A) Msm cells that express Mtb -426 PonA1 show no morphological differences. Scale bar, 2 μm. (B) The Msm cells that only express Mtb -426 PonA1 also double at rates identical to isogenic wildtype, suggesting that -426 PonA1 fully complements growth of Msm that lacks endogenous PonA1. (C) The Mtb PonA1 allele is produced at similar levels to Msm PonA1 (the nonspecific band demonstrates lane 2 has less protein loaded). (D) Mass spectrometric analysis confirms that PknB phosphorylates Mtb MBP-PonA1cyto (with the -426 start site) in vitro. (TIF) [file ppat.1005010.s007.tif]

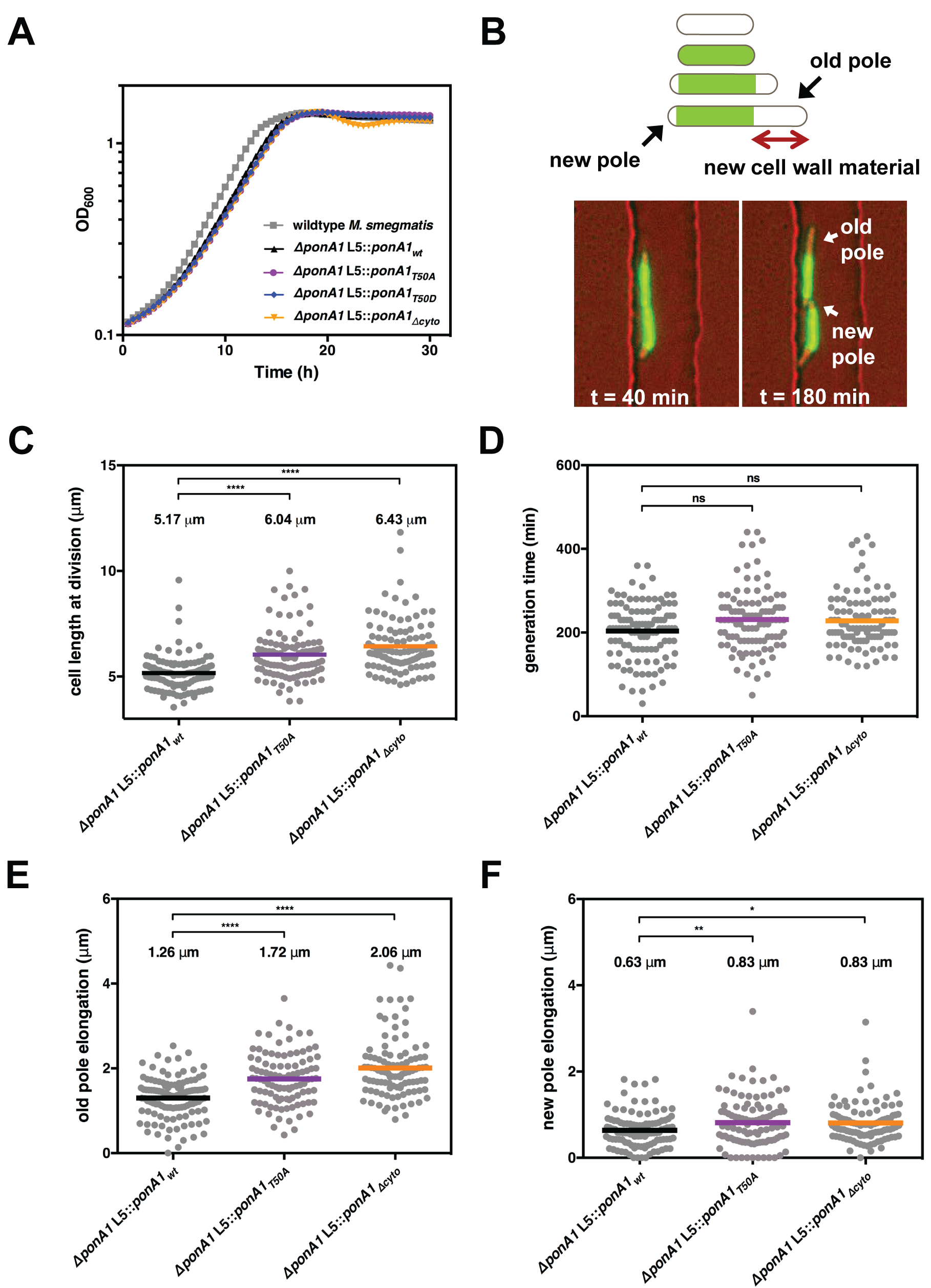

Supplement: S8 Fig — (A) Msm cells that express phosphorylation mutants (T50A and T50D) or a truncation of the cytoplasmic tail of PonA1 (Δcyto) exhibit similar population doubling rates to isogenic wildtype or wildtype Msm. (B) To investigate the impact of PonA1’s phosphorylation on cell elongation and division, cells were stained with a green fluorescent dye that binds to the cell surface. After staining, single cells were imaged in custom microfluidic devices, and new cell wall elongation and division events were measured. (C) The increase in single cell elongation rate of cells that express PonA1T50A correlates with an increased length of single cells at division, as expected. (PonA1wt compared with PonA1T50A approximate p-value < 0.0001 by the Kolmogorov-Smirnov test. PonA1wt compared with PonA1Δcyto approximate p-value < 0.0001 by the Kolmogorov-Smirnov test). (D) Expression of T50A or Δcyto PonA1 do not impact single cell generation times. This suggests that the observed increased cell length is due to faster single cell elongation rates alone and is not impacted by altered septation timing. Significance was assessed by the Kolmogorov-Smirnov test, and neither mutant population was statistically different than PonA1wt. (E) Cell elongation still occurs predominantly from the old pole in the absence of PonA1’s phosphorylation or cytoplasmic tail. (PonA1wt compared with PonA1T50A approximate p-value < 0.0001 by the Kolmogorov-Smirnov test. PonA1wt compared with PonA1Δcyto approximate p-value < 0.0001 by the Kolmogorov-Smirnov test). (F) The new pole exhibits mild increased elongation in cells that express T50A or Δcyto PonA1 compared to wildtype. (PonA1wt compared with PonA1T50A approximate p-value = 0.0036 by the Kolmogorov-Smirnov test. PonA1wt compared with PonA1Δcyto approximate p-value = 0.0261 by the Kolmogorov-Smirnov test). Together with (E), these data suggest that loss of PonA1’s phosphorylation does not impact subcellular distribution of elongation complexes or PonA1’s lo [file ppat.1005010.s008.tif]

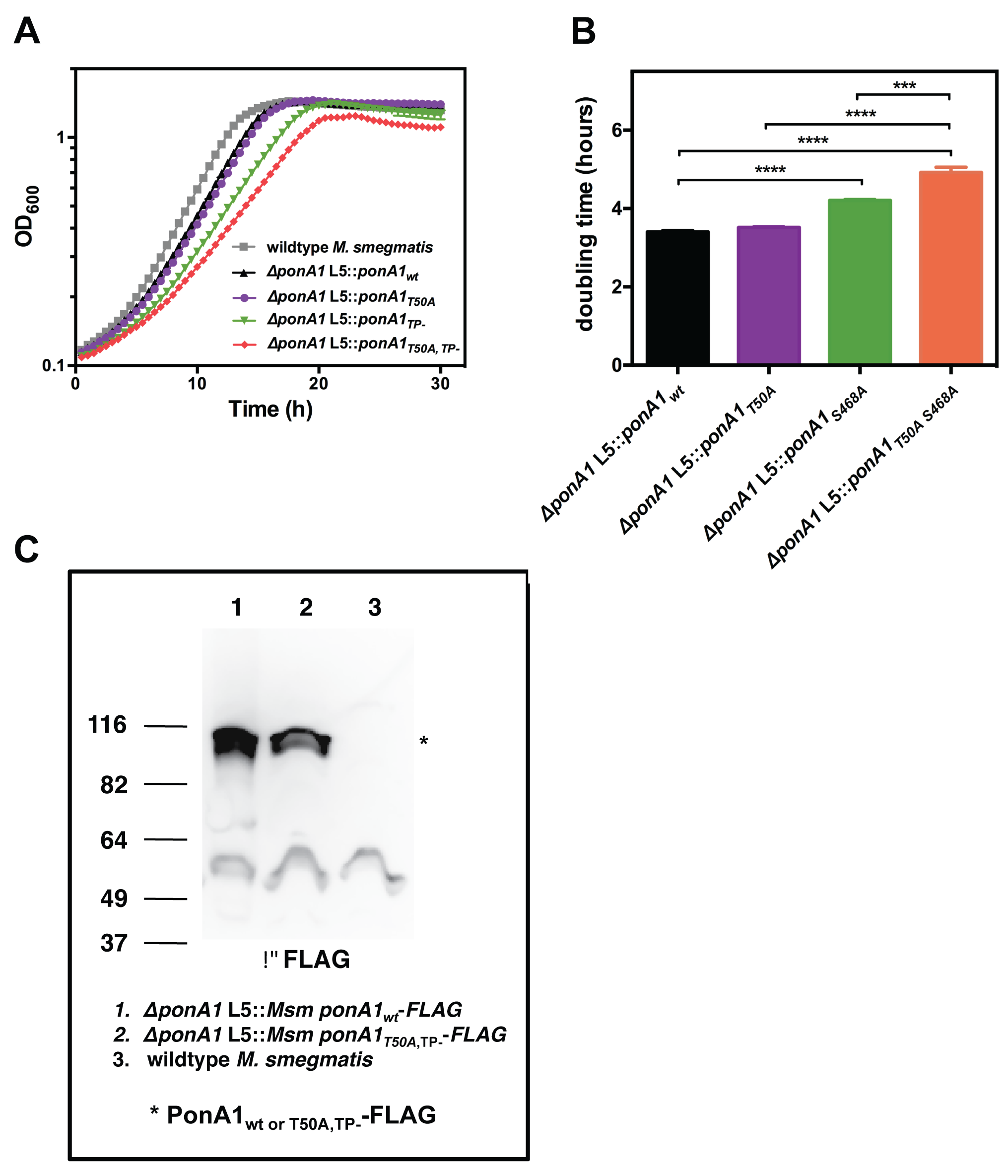

Supplement: S9 Fig — (A) Cells that express a T50A,TP- allele of PonA1 exhibit slower population doubling time as compared to the single point mutants or isogenic wildtype, suggesting that PonA1’s phosphorylation may regulate TG activity to promote normal cell elongation and division. (B) T50A cells double on average every 3.52 hours, TP- cells double every 4.21 hours (p-value < 0.0001 by the unpaired t-test compared to isogenic wildtype), and T50A,TP- cells double every 4.92 hours (p-value < 0.0001 by the unpaired t-test compared to isogenic wildtype; p-value = 0.0008 compared to TP- cells; p-value < 0.0001 by the unpaired t-test compared to T50A cells), whereas isogenic wildtype doubles every 3.40 hours. (C) The T50A,TP- allele is translated into a stable protein. (TIF) [file ppat.1005010.s009.tif]

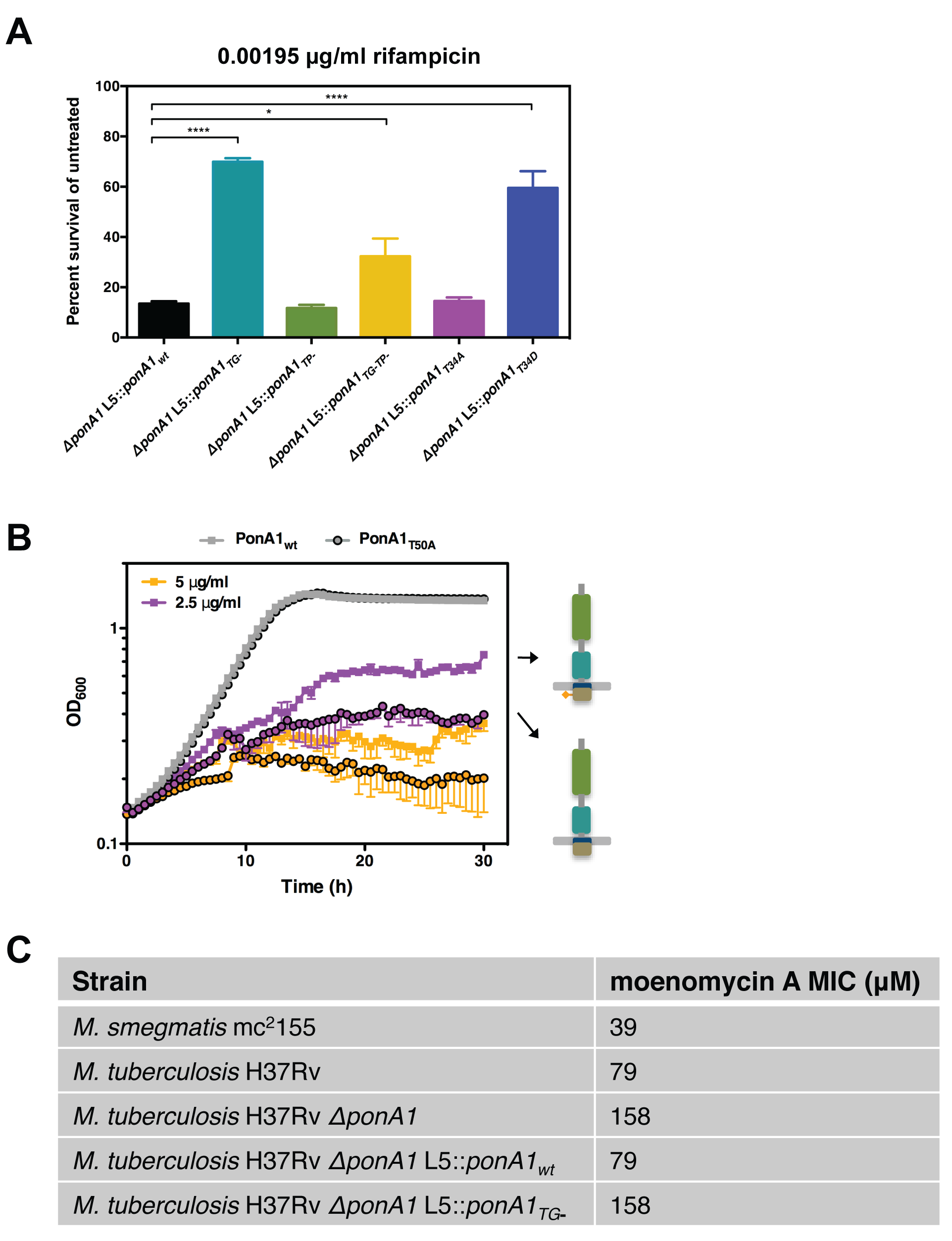

Supplement: S10 Fig — (A) PonA1 single nucleotide polymorphisms identified in clinical isolates have previously been shown to change the tolerance of M. tuberculosis to rifampicin, a frontline tuberculosis therapy. We measured the impact of PonA1 catalytic and regulatory mutants on cell fitness during rifampicin treatment, and found that PonA1TG- and PonA1T34D mutants have 5- and 4-fold increased tolerance to rifampicin compared to isogenic wildtype. Other catalytic (TP-, TG-TP-) or regulatory (T34A) mutations do not alter rifampicin susceptibility. These data suggest that PonA1’s phosphorylation may regulate PonA1’s TG activity and that alterations to PonA1 function impact rifampicin tolerance. (Statistical significance was assessed by one-way analysis of variance with Bonferroni’s multiple comparison test, and multiplicity adjusted p-values are reported. PonA1wt compared to PonA1TG- p-value < 0.0001; PonA1wt compared to PonA1TG-TP- p-value = 0.0295; PonA1wt compared to PonA1T34D p-value < 0.0001). (B) Changes to PonA1 activity also influence cell fitness in the presence of TP domain inhibitors, including those that target both d,d- and l,d-transpeptidases (meropenem). The expression of T50A PonA1 impacts M. smegmatis cell fitness during meropenem treatment, corroborating the importance of normal PonA1 regulatory activity in the maintenance of cell fitness during antibiotic pressure. (C) The TG inhibitor moenomycin exhibits modest efficacy against Msm and Mtb. (TIF) [file ppat.1005010.s010.tif]

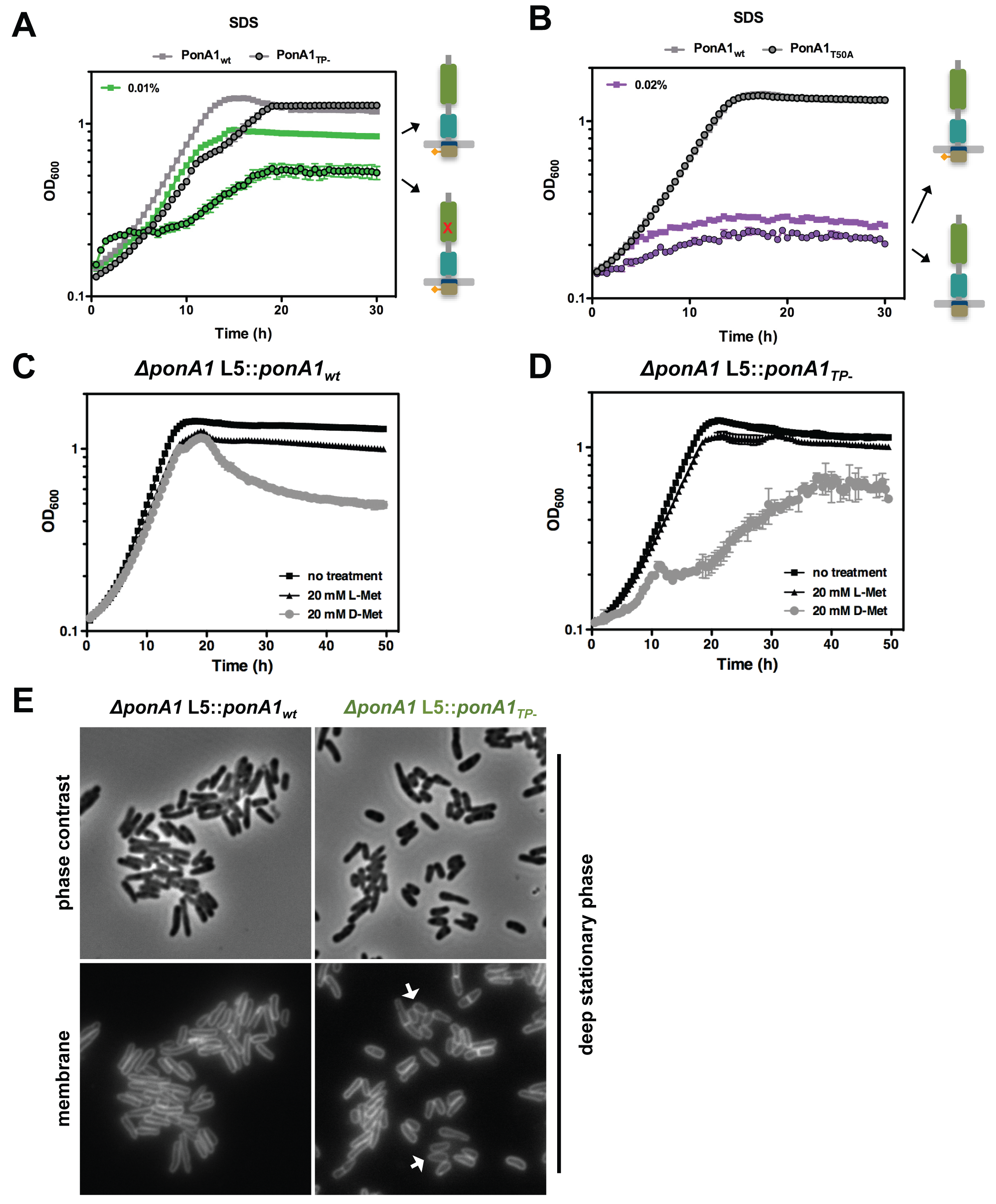

Supplement: S11 Fig — (A) Cells that lack PonA1’s TP activity are less fit during incubation with SDS as compared to isogenic wildtype cells. This suggests that loss of PonA1’s PG crosslinking impinges on cell wall integrity. (B) Cells that express a PonA1 T50A allele also exhibit a modest defect in population doubling in the presence of SDS, suggesting changes to PonA1’s phosphorylation status also impact cell wall integrity. (C) Incubating isogenic wildtype Msm cells in the presence of D-amino acids, which may be incorporated into the cell wall, shows that PonA1’s PG crosslinking may be important for properly incorporating these non-canonical amino acids into the cell wall. (D) Cells that lack PonA1’s TP activity exhibit less robust population growth in the presence of D-Met. (E) Cells that express a TP- PonA1 exhibit cell shape defects when in deep stationary phase (cultured for four days). Cells become wider, shorter and rounder than isogenic wildtype, suggesting that PonA1’s crosslinking is important for PG integrity during stress. (TIF) [file ppat.1005010.s011.tif]
